# Fibril Surface-Dependent Amyloid Precursors Revealed by Coarse-Grained Molecular Dynamics Simulation

**DOI:** 10.3389/fmolb.2021.719320

**Published:** 2021-08-06

**Authors:** Yuan-Wei Ma, Tong-You Lin, Min-Yeh Tsai

**Affiliations:** Department of Chemistry, Tamkang University, New Taipei City, Taiwan

**Keywords:** abeta, MD simulation, coarse-grained model, fibril surface, secondary nucleation, fibrillar twisting, binding sites, elongation (growth)

## Abstract

Amyloid peptides are known to self-assemble into larger aggregates that are linked to the pathogenesis of many neurodegenerative disorders. In contrast to primary nucleation, recent experimental and theoretical studies have shown that many toxic oligomeric species are generated through secondary processes on a pre-existing fibrillar surface. Nucleation, for example, can also occur along the surface of a pre-existing fibril—secondary nucleation—as opposed to the primary one. However, explicit pathways are still not clear. In this study, we use molecular dynamics simulation to explore the free energy landscape of a free Abeta monomer binding to an existing fibrillar surface. We specifically look into several potential Abeta structural precursors that might precede some secondary events, including elongation and secondary nucleation. We find that the overall process of surface-dependent events can be described at least by the following three stages: 1. Free diffusion 2. Downhill guiding 3. Dock and lock. And we show that the outcome of adding a new monomer onto a pre-existing fibril is pathway-dependent, which leads to different secondary processes. To understand structural details, we have identified several monomeric amyloid precursors over the fibrillar surfaces and characterize their heterogeneity using a probability contact map analysis. Using the frustration analysis (a bioinformatics tool), we show that surface heterogeneity correlates with the energy frustration of specific local residues that form binding sites on the fibrillar structure. We further investigate the helical twisting of protofilaments of different sizes and observe a length dependence on the filament twisting. This work presents a comprehensive survey over the properties of fibril growth using a combination of several openMM-based platforms, including the GPU-enabled openAWSEM package for coarse-grained modeling, MDTraj for trajectory analysis, and pyEMMA for free energy calculation. This combined approach makes long-timescale simulation for aggregation systems as well as all-in-one analysis feasible. We show that this protocol allows us to explore fibril stability, surface binding affinity/heterogeneity, as well as fibrillar twisting. All these properties are important for understanding the molecular mechanism of surface-catalyzed secondary processes of fibril growth.

## 1 Introduction

The formation of oligomeric species of Abeta protein and subsequent amyloid deposition are implicated in causing the pathogenesis of Alzhaimer’s Disease (AD) (Chen and Mobley, 2019). As the hallmark of AD, amyloid fibrils display a range of structural variations called fibril polymorphism (Tycko, 2015; Riek and Eisenberg, 2016), which challenges the developments for molecular imaging and therapeutic strategies (Fändrich et al., 2018). The fibrillar structure of amyloid-beta peptides (Abeta40 and Abeta42), for example, are quite different (Colvin et al., 2016; Wälti et al., 2016; Gremer et al., 2017), but they share similar protofilament structures and both display primarily left-handed twisted filament architecture *in vitro* (Schmidt et al., 2009; Zhang et al., 2009). Brain-derived amyloid fibrils, however, are right-handed (Kollmer et al., 2019). These studies demonstrate how amyloid proteins show their structural plasticity under different contexts, and thus are important in determining the pathogenesis of neurodegenerative disorders. Despite many fibrillar morphologies being available, the molecular mechanism underlying the aggregation process of amyloid proteins is still not fully understood. In the process of fibril growth, cross-seeding experiments have revealed some correlation of fibril growth over fibrillar surfaces with selected amyloid peptides. Abeta40 and Abeta42 can cross-seed their constituent fibrils, however, the growth rate displays a different profile for different original fibril seeds (Thacker et al., 2020). Different amyloid peptides can also mutually seed each other. These results suggest a common structural feature of the fibrillar surface that exhibit physicochemical similarity at the molecular level, though identical amyloid backbone virtually is not sufficient for cross-seeding (Daskalov et al., 2021).

Amyloids form by a sequence of chemical reactions. Protein monomers first need to oligomerize into critical nuclei through primary nucleation (Tsai, 2019). These nuclei then may transform into active oligomer species for subsequent secondary processes to occur, for example, elongation, fragmentation, and secondary nucleation. These processes all together make aggregation itself much more complex than descriptions using simple mass-action kinetics (Xue et al., 2008; Tsai et al., 2015). Recent advances in exploring the aggregation free energy landscapes of Abeta peptides have shown the complex paths of interconversion between different but structurally similar states of oligomers and have demonstrated the structural diversity for conformational conversion between pre-fibrillar to fibrillar oligomers (Zheng et al., 2016; Zheng et al., 2017). The detailed molecular interactions such as salt bridges, intercalation of water molecules, and hydrophobic clusters formed in different fibril polymorphic forms were found to significantly affect the capacity for cross-seeding as well as secondary nucleation (e.g., speeding up the aggregation of Abeta40 with Abeta42 fibril) (Xiao et al., 2015; Colvin et al., 2016). These results suggest the role of early stages of aggregation in modulating the chemical properties of the fibrillar surfaces—surface heterogeneity—that provide different specific or nonspecific chemical screening capacity. Surface heterogeneity, perhaps, plays a pivotal role in the later stages of aggregation that involves a variety of secondary processes.

To understand the molecular mechanism underlying surface-dependent aggregation events, in this work, we focus on the formation of monomeric precursors on the existing fibrillar surfaces as it can potentially initiate subsequent secondary processes. There are several possibilities when a protein monomer interacts with an existing fibrillar surface. During the interaction process, the monomer can either bind and stay at a particular surface patch, forming a new surface nucleation site or the monomer ends up localizing at both ends and elongates the fibril as a result. The former process, secondary nucleation, describes protein monomers being nucleated on the fibrillar surface through a two-dimensional search. This newly formed critical nucleus then plays a role of seeding new fibrils. The latter describes the elongation of the existing fibrillar structure which may take place at both fibril ends along the fibril axis. Although “elongation” and “secondary nucleation” are two seemingly distinct aggregation processes, it is now a popular view that they should be investigated with caution at the molecular level, as these two processes can be mutually correlated from a broader view of aggregation energy landscapes Cohen et al. (2018); Scheidt et al. (2019).

Elongation is the major process of aggregation when growing a fibril. There exist considerable experimental studies on the elongation process, in particular in measurements of the rate of fibril elongation (Xu et al., 2019). The experimental work offers an opportunity for theorists to construct models to understand molecular mechanisms of fibril growth. Some have proposed models concerning structural rearrangements and intermediates, while others address the molecular species from an energy perspective. Wei et al. first carried out atomistic molecular dynamics simulation to study thermodynamics and kinetics of fibril elongation of Abeta17–42. They used a kinetic network model to reveal detailed pathways for fibril elongation (Han and Schulten, 2014). Although elongation primarily concerns monomer addition at fibril ends, kinetic analyses have suggested multiple steps are involved, from solution free monomers all the way to final elongated fibril. Crespo et al. showed that elongation includes lateral migration of attached monomers towards the fibril ends and this process is not rate-limiting (Crespo et al., 2012). Since there exist several polymorphs of fibrils, some are disease-relevant, dissociation of Abeta monomers from such fibrillar structures have also been investigated in silico. For example, S-shaped fibrillar structure showed the stop-and-go mechanism at fibril ends due to the structural flexibility of the N-terminal monomer (Ilie and Caflisch, 2018). Simulation study over a three-fold protofibril from human tissue, however, supported the two-step dock-and-lock mechanism, where Abeta monomer interacts with fibril surface by direct docking onto it, and then, the docked peptide undergoes conformational arrangements on the surface in order to fit the fibril template over the ends for elongation (Sasmal et al., 2016). According to their result, docking is faster than the locking process by about an order or so, depending on the type of monomer ensemble. In contrast to the stop-and-go kinetics, recent experimental work showed relatively steady Abeta40 fibril growth and dissolution rates (Xu et al., 2019). Structural and dynamics difference between the two ends (even/odd) of amyloid fibril adds more kinetic complexity for the understanding of the mechanism of fibril growth. The even end grows faster as has been shown in experiment (Konno et al., 2020), independently verified in another simulation work (Okumura and Itoh, 2016). All these results raise an issue about the pathway-dependent nature for elongation, in particular, due to their different mechanistic details. For example, there might be multiple pathways of elongation channels that can potentially contribute to fibril growth. In this regard, the most probable path has been discussed (Rodriguez et al., 2018). From a kinetic perspective, indeed, multiple pathways would lead to a variation in predicting kinetic properties. Regarding the binding thermodynamics, several approaches are available in the calculation of the binding affinities, for example, alchemical free energy perturbation method or potential-of-mean force (PMF) approach (Deng and Roux, 2009). Depending on the simulation type, different approaches in general yield at least qualitative agreement across different all-atom and coarse-grained force fields.

In contrast to fibril elongation, secondary nucleation describes a surface-catalyzed nucleation process where new fibril seeds emerge. Recently, an experimental study that combines theoretical analysis showed that secondary nucleation and elongation occur at different sites, suggesting a potential dynamic interplay for a protein monomer searching over fibrillar surfaces (Scheidt et al., 2019). In the propagation of amyloid fibrils, researchers have shown the role of hydrophobic patches in growing fibrils *via* secondary nucleation (Thacker et al., 2020). All these studies have clearly pointed out the importance of surface heterogeneity for different secondary processes.

There are many existing studies that focus on amyloid aggregation, either from a nucleated-polymerization perspective or a templated fibril growth perspective. However, little is known about how fibril polymorphic surfaces affect secondary processes. Specifically, how existing fibril seeds catalyze the process of fibril growth by recruiting new monomers onto the fibrillar surfaces. As we have mentioned above, the mechanistic details of aggregation, in particular the kinetically relevant events, are significantly limited by the experimental means and physical/chemical parameters we used to probe them. Theoretical models and simulation techniques, in this regard, are very useful for probing complex processes and therefore allow us to explore parameter space that is difficult to achieve *via* experimental methods alone.

Fully atomistic modeling has shown great promise in tackling many important problems in protein biophysics. This technique is particularly useful for exploring full dynamics of Abeta monomers to necessary atomistic details, and is an ideal approach for exploring conformational ensembles for various calibration purposes (Grazioli et al., 2019). However, modern pressing biological problems involve molecular assemblies having thousands of amino acid residues and functional dynamic motions taking place in timescales more than milliseconds, seconds and beyond. As a result, exploring the biologically relevant timescales using fully atomistic simulation makes such realization a daunting task. Coarse-graining therefore becomes a conceptual prerequisite for addressing the major problems of modern biology. Another important motivation for developing coarse-grained modeling is that many large-scale protein motions concern emergent properties caused by collective organizing principles (Laughlin et al., 2000), in which details of intra/intermolecular forces are averaged out. Folding, binding, and functional transition in proteins are examples of emergent phenomena that can be fully understood at an appropriately coarser resolution. In this study, we use one such coarse-grained model, Associative-memory, Water-mediated, Structure and Energy Model (AWSEM) (Davtyan et al., 2012; Tsai et al., 2016b), to study the process of Abeta peptides interacting with the surfaces of a protofilament. AWSEM is a transferable, coarse-grained, and non-additive protein force field that incorporates physically motivated energy terms and knowledge-based information using the principle of minimal frustration (Ferreiro et al., 2018). AWSEM has been proven useful for exploring many of the important biological processes, such as folding, binding (Tsai et al., 2016b; Zheng et al., 2012), aggregation (Zheng et al., 2016, 2017; Chen et al., 2016), protein-DNA interaction (Tsai et al., 2016a; Tsai et al., 2019; Potoyan et al., 2016a; Potoyan et al., 2016b), and chromosome remodeling (Zhang et al., 2016), and has continued to be a suitable coarse-grained model for studying the aggregation system of interest here. Here, we employ a GPU-enabled AWSEM code, openAWSEM (Lu et al., 2020). This new version includes a recent advance in GPU acceleration built on the openMM platform (Eastman et al., 2017).

In this work, we explore the experimentally determined fibrillar structure of Abeta11-42 (ssNMR) using openAWSEM. We first study the stability of the fibrillar surface structure using the AWSEM coarse-grained force field and confirm the structural integrity of the S-shaped polymorphic fibrillar structure. We then investigate fibrillar surface heterogeneity by exploring the binding free energy landscapes of a free Abeta monomer to a short fibrillar surface. For a broader surface sampling purpose, we choose to use the biasing coordinate that allows efficient sampling over the surface with a simple distance restraint. This biasing strategy is somewhat different from the conventional approach, where the fibril ends usually are chosen to bias with (Han and Schulten, 2014) or some positional restraints at the filament tips are applied in order to prevent twisting motions (Schwierz et al., 2016). These approaches are important for obtaining structurally stable binding sites for elongation. We nevertheless explore the PMF along the coordinates that allow more sampling over amyloid structural precursors that can lead to different aggregation pathways. To ensure this, different initial spatial orientations are used. The biasing coordinates are determined by a C-alpha in the free monomer and a C-alpha of a fibrillar monomer in the fibril (chain in the middle of the fibril, not the fibril ends). We aim to explore the configurations of monomeric amyloid precursors that precede elongation and other secondary processes. Our overall hypothesis is that secondary processes, such as elongation and secondary nucleation, share similar monomeric amyloid precursors that drive different aggregation pathways. We have identified several structural precursors and potential surface binding sites accordingly. To understand the intrinsic energetics of the local residues forming binding pockets, we have carried out energy frustration analysis for a series of fibril polymorphic structures and have predicted the preferred surface binding sites. The simulation protocol used allows us to characterize some key steps in the process of aggregation and allows efficient sampling for binding sites that are specific to secondary processes. Finally, we model the fibrillar twist polymorphism of a protofilament with different filament sizes. We find a filament size dependent effect on filament twisting, which potentially can modulate the surface heterogeneity of a fibril.

Understanding the molecular mechanism of elongation and secondary nucleation can help predict how mutations and external factors affect fibril growth and how antibody drugs intervene in the processes of elongation and secondary nucleation. It allows us to predict the effect of drugging in promotion or inhibition of fibril growth. Our results support that secondary nucleation and elongation occur at different binding sites, confirming their independent inhibitory effects by molecular chaperones follow a different pathway (Scheidt et al., 2019). We anticipate that our study will be useful for developing rational design principles for new therapeutic drugs.

## 2 Methods

### 2.1 Models and Simulation

#### 2.1.1 Modeling of Fibrillar Structure

Human amyloid-beta (Abeta) peptide (pdbID 2MXU [Xiao et al., 2015)] was used to model the aggregation system in the present study. The solid-state NMR structure features a S-shaped, three-layer fibrillar architecture with 12 Abeta peptides (11–42, length = 32), in-register and parallel aligned, labeled as chain A, B, … , L, respectively. In simulation, we prepared one such 12-monomer fibril structure and one additional free monomer having its initial structure as it is in the fibril structure. For the central fibril, “single fragment memory” was used in order to strongly bias the aggregation energy landscape towards the native fibril structure. For the additional free monomer, we adopted two different monomer structural ensembles: 1. Relaxed ensemble (no biasing fragment memory is used) 2. Fibril-like ensemble (single fragment memory as is in the fibril structure). The descriptions about the “fragment memory” library can be found elsewhere (Davtyan et al., 2012; Tsai et al., 2016b). The free monomer is initially positioned in six different orientations with respect to the central fibril: up, down, left, right, front, and back. These six independent simulations ensure a better sampling quality while leveraging the availability of our computation resources. The system is built and visualized using VMD (Humphrey et al., 1996).

#### 2.1.2 Molecular Dynamics Simulation Using OpenAWSEM

In this study, we use openAWSEM, a python version of the AWSEM protein coarse-grained force field developed by Wolynes and his coworkers Lu et al. (2020). This new simulation platform is built on openMM (Eastman et al., 2017) for a fast (GPU-enabled), flexible, easy-to-use purpose. OpenAWSEM inherits from the lineage of the Associative-memory, Water-mediated, Structure and Energy Model (AWSEM), for molecular dynamics (MD) simulation (Davtyan et al., 2012). In AWSEM, each amino acid residue is represented by three atoms: *C*_*α*_, *C*_*β*_, and *O* (glycine is an exception). The physicochemical properties of different types of side chains are reflected on *C*_*β*_ atoms. The AWSEM-MD simulation protocol has been used to address a variety of biological questions, such as protein structure prediction (Davtyan et al., 2012; Tsai et al., 2016b; Sirovetz et al., 2017; Chen et al., 2018a), protein binding prediction (Tsai et al., 2016b), protein aggregation (Zheng et al., 2016, 2017), as well as complex protein-DNA assemblies and remodeling (Tsai et al., 2016a, 2019; Potoyan et al., 2016b; Zhang et al., 2016). Interested readers are encouraged to test the online web-server version, AWSEM-Suite for structure prediction (Jin et al., 2020). Because of openMM’s extensibility in python scripting, openAWSEM benefits from such flexibility for interfacing with other post-processed analysis and visualization toolkits, such as MDTraj, pyEMMA and NGLviewer.

For the fibril stability test, three independent simulations were carried out, with each simulation trajectory running for 10 million simulation time steps (=10,000 frames).

#### 2.1.3 Importance Sampling and WHAM

Free energy calculation, more precisely potential of mean force (PMF), is carried out using the pyEMMA python package, developed by Noe and his coworkers (Scherer et al., 2015). In the calculation of PMFs, one needs to choose a specific progress coordinate of interest to sample along. Because high energy configuration space is not easily accessed through thermal activation, a biasing force is required in order to “bias” the sampling route towards the configurational space of interest-a procedure termed “importance sampling”. In practical use, all the sampling tasks were carried out by running molecular dynamics simulation on the openAWSEM platform (Lu et al., 2020). To sample along the route of a free monomer diffusing towards a designated position on the surface of a fibril, a large number of sampling windows were prepared. Different sampling windows were deployed by applying a series of harmonic biasing restraints between two atoms (one from the free monomer and the other from the fibril); they are centered at a distance in a range of 10–100 Å (with an interval of 1 Å). A total of 91 independent simulations were generated as a result. The biasing coordinate is defined as the distance between the C-beta atom of residue N27 of chain F in the fibril and the C-beta atom of residue N27 of the free monomer. The biasing force constant k is set to be 2.4 kcal/mol (10 kJ/mol) for all the 91 simulations (sampling windows) using the harmonic biasing form $k{\left(r-r_{0}\right)}^{2}$ , with *r*_0_ = 10, 11, 12, … 100 Å. Each simulation is run for 5 million time steps; 5,000 frames were outputted for analysis purposes (output frequency is every 1,000 time steps). After collecting data from the simulation trajectories (from a total of 91 sampling windows), the data were reweighted using the WHAM technique (Kumar et al., 1992) implemented in pyEMMA package (thermo.wham) (Scherer et al., 2015), discarding the first 500 thousands time steps (500 frames) for equilibration. The distribution of all the sampling windows is shown in the Supporting Information (Supplementary Figures S1, S5).

### 2.2 Analyses

#### 2.2.1. Thermodynamic Binding Affinity

**FIGURE 1:**
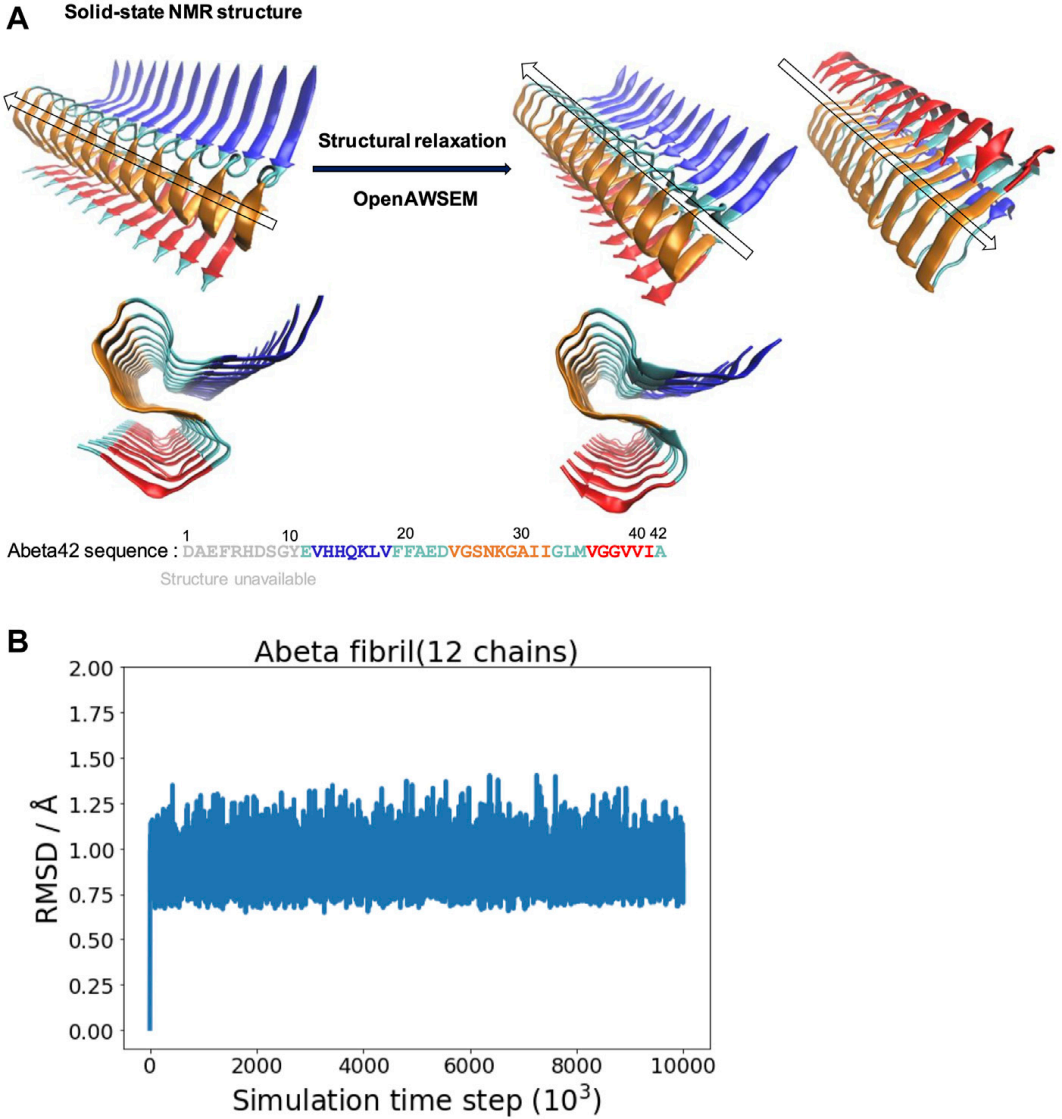
The overall structure of the experimentally determined fibrillar structure and the simulated fibrillar structure are compared. **(A) Left:** The fibrillar structure of Abeta42 is determined by solid-state NMR [PDB ID: 2MXU (Xiao et al., 2015)]. The actual sequence length of the individual peptides in the structure is 32 (spanning from 11 to 42). The “S” shaped triple parallel-beta-sheet architecture can be seen from the side view below. **Right:** The simulated fibrillar structure. The experimental structure is structurally relaxed *via* molecular dynamics simulation using openAWSEM. Two different orientations of the relaxed structure are shown, with an arrow showing the direction of the fibril axis. The “S” shaped triple parallel-beta-sheet remains in the simulation. Different colors represent different local structural features of the Abeta42 peptide, defined in the original PDB file, illustrated using the sequence below. Blue strand (V12-V18), Cyan loops (E11,F19-D23,G33-M35,A42), Orange strand (V24-I32) and Red strand (V36-I41). **(B)** The RMSD is calculated as a function of simulation time step (only one of the three independent simulation trajectories is shown).

In the calculation of binding affinity, we use the formula that is based on one-dimensional radial potential of mean force (PMF) given in the literature (Deng and Roux, 2009). One simple equation is shown below.

$$K_{d}=4\pi \int_{site}r^{2}e^{-\beta \left(w\left(r\right)-w\left(r^{*}\right)\right)}dr$$ (1)

where *w* refers to the PMF as a function of the distance *r*; r* refers to a reference position set to be far away in the bulk (*w*(r*) = 0 at r* = 80 Å); *β* = 1/*k*_*B*_ *T*. In practical use, to enhance the fluctuations of the orientation of the Abeta monomer (ligand) with respect to the central fibril, we adopted six independent simulations with each of them representing a different initial orientation (front, back, up, down, even, odd). See the subplot in Figure 1A. The thermodynamic binding affinity thus can be calculated

$$\Delta G{}^{\circ}=-RT\ln\left(c_{0}K_{b}\right),$$ (2)

where *c*_0_ = 1*M* refers to the standard state (1 mol/L = (1,660 Å^3^)^−1^). See Table.1 for the calculated value as well as the experimental values obtained from literature.

**TABLE 1:** Thermodynamic binding affinity of Abeta binding to a fibril.

∆Gb (kcal/mol)	Top	Length	Solubility (µM)	Condition	Type
−8.7	Twofold	1–40	0.44	<10 µM (27°C)	Exp. Xu et al. (2019)
−8.7	Twofold	1–40	0.3–0.4	<75 µM (24°C)	Exp. Qiang et al. (2013)
−9	—	1–40	0.8–1.0	<30 µM (37°C)	Exp. O’Nuallain et al. (2005)
−12 (even)	Twofold	17–42	—	37°C	Sim. Han and Schultan, (2014)
−11.3 (odd)					
−15.6	Twofold	9–40	—	37°C	Sim. Schwierz et al. (2017)
−25.8	Single	11–42	—	27°C	This Work

#### 2.2.2 Probability Contact Maps

The detailed monomer-fibril interactions are quantified using the Contact Map Explorer python module, which is based on tools implemented in MDTraj (McGibbon et al., 2015). The formation of a contact is defined by setting a cutoff distance between any of two C-alpha atoms in the residues. A contact forms if the distance is smaller/equal to the cutoff value; the cutoff is set to be 6.5 Å in the present study. The step-by-step tutorial (Jupyter notebook) is provided in the Github.

#### 2.2.3 Nematic/Polar Order Parameter

To characterize the structural difference of fibril polymorphism, we have used the nematic order parameter (*P*_2_ value) as the structural orderness of fibrillar surface structure. Originally designed for describing the structural order of liquid crystals, nematic order parameter was first introduced to study protein aggregation by Caflisch and co-workers (Cecchini et al., 2004). This order parameter was further applied to describe elongation of Amyloid-beta fibrils (Schwierz et al., 2017) and recently being compared with neural network learned order parameters Charest et al. (2020).

#### 2.2.4 Probing Fibrillar Twisting

The twist angle is calculated using the protocol described in the literature (Ilie and Caflisch, 2018). The angle, *θ*_*i*_, is defined by the two vectors: one represents the vector of the reference chain *i* and the other denotes the vector of the neighboring chain *i* + 1, respectively (see Figure 6A). Similarly, *θ*_*i*+1_, *θ*_*i*+2_ and so forth can also be calculated by propagating the current chain pair to the next neighboring chain pairs. The vector is defined by the two C-alpha atoms of Q15 and F19 of individual chains as indicated in the reference (Ilie and Caflisch, 2018). To avoid the effect of simulated fibril structural fluctuation on angle calculation, we defined a fibril axis vector and a normal plane perpendicular to this vector for angle correction purpose. We report two different angular properties in order to quantify the twist morphology of a fibril. 1. The averaged *θ* ( $\theta^{¯}$ , the twist angle is averaged over all chain pairs of the same fibrillar structure) 2. The accumulated total twist angle, *θ*_*tot*_ (the twist angles from all of the chain pairs are added up). *θ*_*tot*_ represents the extent of global twisting morphology of the fibril.

In simulating fibrillar helical twisting, five protofilament models of different sizes (12, 24, 36, 48, 62 chains) were prepared. Long protofilament models (24–62 chains) were made using the 12-chain model (pdbID 2MXU). To ensure the strands at the joint boundary are properly connected between the 12-chain model fragments, we also, if necessary, constrain the resulting elongated protofilament by applying a mild harmonic biasing force along the fibril long axis. The harmonic biasing form is $\frac{1}{2}k^{\prime }{\left(r-r_{0}\right)}^{2}$ .The magnitude of the force constant/center distance pairs (*k*′, *r*_0_) were set to be (6 kcal/mol, 10 Å). The center of mass of the first two chains and the center of mass of the last two chains of the model fragment were the constrained objects to which the biasing force is applied. As a result, we carried out 1 million simulation time steps for all the five protofilament models for a pre-equilibration/relaxation purpose. The relaxed structure of each protofilament model along with the structure’s *θ* angle distribution can be found in the Supporting Information (Supplementary Figure S10). After that, 10 million simulation time steps were performed and the data (each with 10,000 frames) were collected for fibrillar twisting analysis.

#### 2.2.5 Frustration Profiles for Polymorphic Fibrillar Surfaces

According to the energy landscape theory of protein folding, the evolutionarily conserved protein structure is energetically minimally frustrated while protein functional activities emerge through frustration (Ferreiro et al., 2018). The corresponding energy landscape for robust folding is manifested as a funneled shape. However, a recent study has pointed out functional roles of energetically frustrating areas in binding protein-DNA partners (Tsai et al., 2016b,a; Marcovitz and Levy, 2013; Potoyan et al., 2017), forming assemblies, and ligand binding (Chen et al., 2020). Energy frustration of proteins involves the statistical energy survey over a series of decoy states, which can be generated through pairwise residue substitution, direct mutation, and position shifting. Different decoy settings correspond with different physical contexts. The frustration is defined using the standard scores (z-scores) in statistics. Three different scales are classified accordingly: Frustrated (<−1), neutral (>−1 and <0.78), and minimally frustrated (>0.78). Interested readers should refer to the reference provided for details (Parra et al., 2016).

The frustration calculation for a series of amyloid fibrillar surfaces was conducted using the frustratometer server Parra et al. (2016), http://frustratometer.qb.fcen.uba.ar/. This frustration computation protocol utilized the same AWSEM-MD energy function (sequence separation is set to be 3) along with the electrostatics enhanced feature (optional) to compute the frustration profile. The frustration profiles for a variety of polymorphic fibrillar structures are shown in Figure 5. The PDBIDs used in the analysis include 5KK3 5OQV 2MXU 2M4J 2LMQ 2LMN. The results are summarized in Table 2. The protein residues in purple indicate they are conserved residues and therefore energetically minimally frustrated, while the residues in red represent highly frustrated residues. This means they may have functional significance in interacting with its partner, such as protein, DNA/RNA, or even membranes.

**TABLE 2:** Polymorphic properties of aggregation.

	Polymor.1	Polymor.2	Polymor.3	Polymor.4	Polymor.5	Polymor.6	This work
PdbID	5KK3	5OQV	2MXU	2M4J	2LMQ	2LMN	2MXU
# of	4	6	4	6	4	4	2
patches							
Frustrated	E11	D1,E3	E11	D1,E3	Y10,E11	Y10,E11	E22, D23
residues	H14,Q15	E11	K16	E11	K16	K16	N27, K28
	K16	Q15,K16	E22,D23	K16	E22,D23	E22,D23	
	E22,D23	E22,D23	N27,K28	E22,D23	G25,S26	S26,N27	
	N27,K28	N27,K28		K28	N27,K28	K28	
*P*_2_ value	0.95	0.93	0.99	0.25	0.26	0.93	0.99
Method	ssNMR	cryoEM	ssNMR	ssNMR	ssNMR	ssNMR	Sim

## 3 Results and Discussion

### 3.1 The Same “S” Shaped, Triple Parallel-Beta-Sheet Architecture Remains in the Simulation

Starting with the experimentally determined fibrillar structure, we examine the stability of the fibrillar structure in the simulation using the AWSEM force field. We find that the overall fibrillar structure along with its surface architecture is well maintained in the simulation. Figure 1A shows the structural refinement of Abeta42 fibrillar structure. The NMR structure exhibits a S-shaped triple parallel-beta-sheet architecture. This fibrillar architecture shows a common cross-beta structure, which contains specific cooperative residue interactions along the 2D plane that is perpendicular to the fibril axis. Figure 1B shows one of the three RMSD trajectories (based on the Root Mean Square Deviation of *C*_*α*_ atoms with respect to the reference structure of 2MXU); the rest of two are not shown. The RMSD value is saturated at an averaged value of 0.90 Å; the average is taken over the last 1,000 frames. The resulting polymorph has been described as “ribbon folding” by Wolynes and his coworkers (Chen et al., 2018b). Because of the cooperative coupling among different fibril dimensions, a variety of fibrillar polymorphs become possible when different pairs of residues are preferred using different force fields. According to the ribbon-folding landscape schemes, different fibrillar architectures can be seen at the energy local minimum along the polymorph energy landscape (Chen et al., 2018b). In this study, we show that openAWSEM is suitable for exploring the ideal cross-beta fibrillar architecture. The cross-beta architecture along with its fibrillar surface later will be used to study surface heterogeneity and helical twisting of a protofilament. Here, we show that the openAWSEM-refined fibrillar structure retains the same S-shaped, triple parallel-beta-sheet architecture observed from the NMR structure.

### 3.2 A Monomer Binding to Fibrillar Surfaces can be Characterized at Least by Three Different Stages: Free Diffusion, Downhill Guiding, and Dock and Lock

**FIGURE 2:**
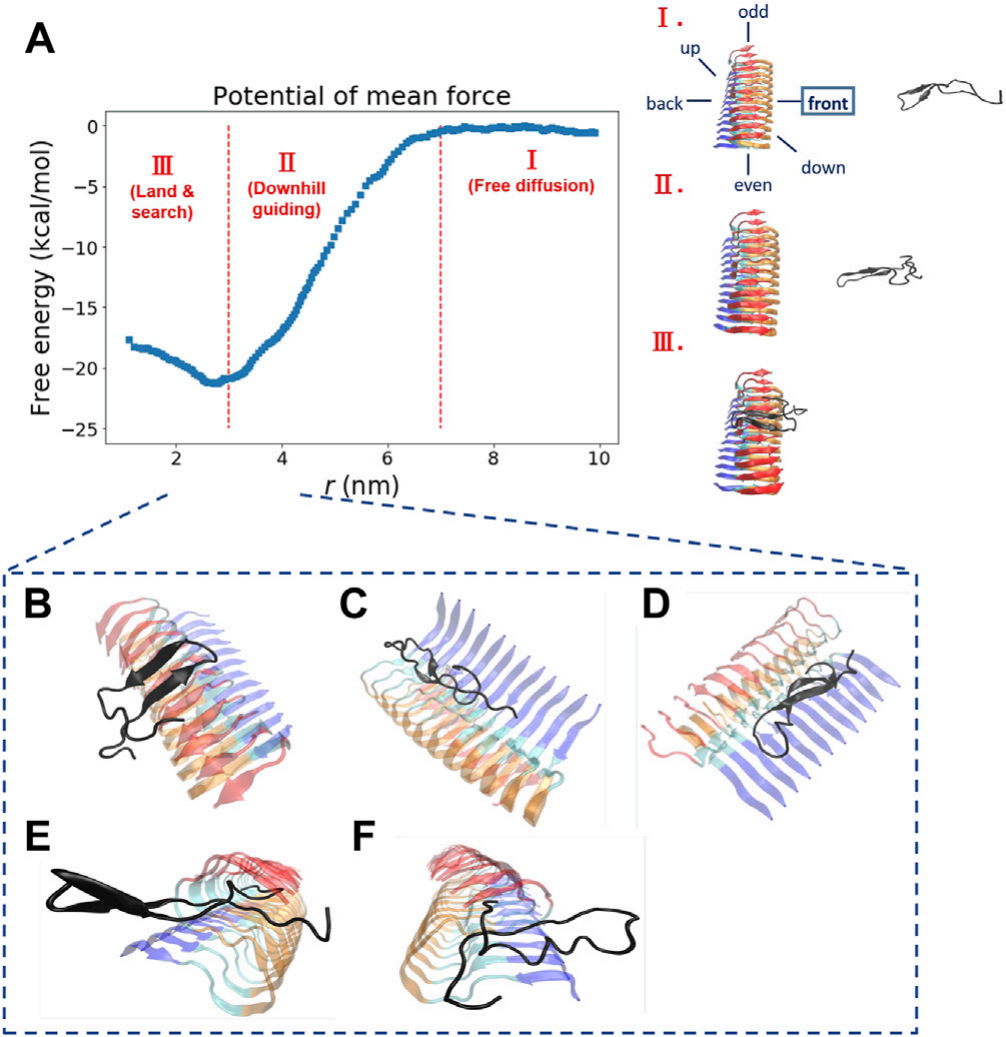
The free energy profile for a single Abeta11-42 monomer binding to the Abeta42 fibrillar surface (12 chains) is shown. **(A)** The free energy profile features three different aggregation stages, labeled as I. Free diffusion. II. Downhill guiding. III. Dock and lock. *r* is defined as the distance between the C-beta of residue 27th in the free monomer and the C-beta of residue 27th of chain F in the fibril. A representative configuration at each aggregation stage is schematically shown on the right. Simulations were prepared with six different monomer positions with respect to the central pre-existing fibril. Six orientations: front, back, up, down, even, odd are schematically shown in the diagram. The free energy profile shown refers to the result obtained from the simulation setup with the monomer positioned in the “front” orientation. The free energy profiles for the rest of the orientations are shown in the Supporting Information (Supplementary Figure S2). **(B–F)** The representative structures taken from stage III in which different binding configurations are formed upon the monomer landing and searching over the fibrillar surface. These binding configurations are potentially surface-catalyzed precursors for fibril growth. **(B)** C-ter surface precursor **(C)** N-ter surface precursor **(D)** Cleft-gate precursor **(E)** Even-end precursor **(F)** Odd-end precursor.

We explore the free energy landscape along a distance separation between a free Abeta monomer and a fibril surface. To enhance sampling over different spatial orientations, we carry out several independent simulations with different initial positions of the monomer with respect to the central fibril. A total of six different positions were chosen to address the fluctuations of orientation. The six simulations, having the monomer being put in different orientations: front, back, up, down, even, and odd, respectively, were performed (see the subplot in Figure 2A for a schematic description). Figure 2A presents a representative free energy profile with the monomer being positioned in the “front” position. From the free energy profile, several features can be observed. They are classified into three different stages accordingly: I. Free diffusion. II. Downhill guiding. III. Dock and lock. When the Abeta monomer is far from the central fibril (*r* > 80 Å), the dynamics of the free monomer is primarily diffusive and that the free energy profile is nearly flat in the plateau (Stage I). As the distance between the fibril and the monomer decreases, the monomer is subject to a long-range guiding force due to electrostatics, and therefore, the monomer begins to approach the fibril. This long-range guidance yields an energetically downhill profile (Stage II). The downhill free energy continues until its slope significantly changes at *r* ≈ 30 Å where the free energy profile displays a curvature. After that, the monomer begins to have physical contacts with the fibril (Stage III). In stage III, there are many ways for the monomer to dock the fibril. The biasing strategy used allows spatially orientational flexibility for the monomer to dock the fibril. As a result, the monomer is able to dock the fibrillar surface through different sites. All the resulting binding configurations lead to a clear free energy basin at *r* ≈ 30 Å. Figures 2B–F show example configurations of several key binding configurations whose population is significant and that their interaction pattern is well characterized. The result shows that the monomer can interact with the fibril’s C-terminal surface (red), N-terminal surface (blue), cleft interface, even-end, and odd-end. We will look into their structural features more carefully in the next section. The rest of the free energy profiles, with the monomer initially positioned in a different orientation, can be found in the Supporting Information (Supplementary Figure S2).

Next, we look into the thermodynamic binding affinity, defined by the potential of mean force (PMF). In determining the free energy of binding, multiple free energy calculations have shown variation in *r*_*b*_ (*r*_*b*_ refers to the distance at which the global free energy basin is found), suggesting that the Abeta monomer binds to the fibril surface through a pathway-dependent manner. This pathway dependence very likely causes some variations in the binding free energy profiles since the monomer might interact with the fibril surfaces through different “dock” sites. Here, we do not assume any specific binding site *a priori* for the monomer to bind with. Instead, we aim to sample different binding trajectories and then combine these trajectories to determine the standard binding affinity (with *c*_0_ = 1 M, see Section 2.2.1). The value is computed to be −25.8 ± 2.4 kcal/mol if we use the data of all the six orientations to ensure the orientational fluctuations. The binding affinity, determined by the simulation trajectory of individual single orientation, ranges from −23 to −29 kcal/mol. This energy variation is due to a different PMF obtained from individual orientation (see Supporting Information for details). The experimental values of −8.7 kcal/mol (Xu et al., 2019; Qiang et al., 2013) and −9 kcal/mol (O’Nuallain et al., 2005). have been exclusively reported for the process of fibril elongation. Their corresponding binding affinity was also calculated using computational methods, which are summarized in Table 1 as well. Although the reported values for binding affinity are rather diverse, these values are within the same order of magnitude. Fibrillar surface heterogeneity, presumably, plays an important role in the process of monomer binding. As a result, finding a proper reaction coordinate is a non-trivial task. In other words, monomer binding may undergo different pathways; the overall process can be under a kinetic control. For example, when the monomer binds to a specific surface site, the monomer may undergo a conformational conversion, searching for the right conformation or the position for subsequent secondary events. Indeed, several surface-dependent aggregation mechanisms have been discussed, such as conformational rearrangement (Xu et al., 2019), lateral migration (Crespo et al., 2012), or other surface-based events. These all together may have significant influence on the thermodynamic interpretation of binding and thus determine the kinetics of fibril growth. A similar multi-pathway issue using a complex collective variable for describing the loop interaction in adenine riboswitch has been discussed in the literature (Di Palma et al., 2015). It is important to know if the progress coordinate of interest is sufficient to drive the system through the appropriate transition states. Here, we recall the importance of the biasing protocol used for the interpretation of results.

One interesting result is worth noting. Free energy calculation using a different monomeric structural ensemble (fibril-like) shows a somewhat similar free energy profile (with the same three stages as described above) but now with a rather different pattern of contacts (see next section for further discussion). This result can be attributed to the structural rigidity of the specific fibril-like conformation used for the monomer. This finding suggests that the specificity for binding energy are encoded in the sequence, irrespective to the monomer conformation adopted.

### 3.3 Surface Binding Heterogeneity: Several Binding Sites are Identified Over the Fibrillar Surfaces

**FIGURE 3:**
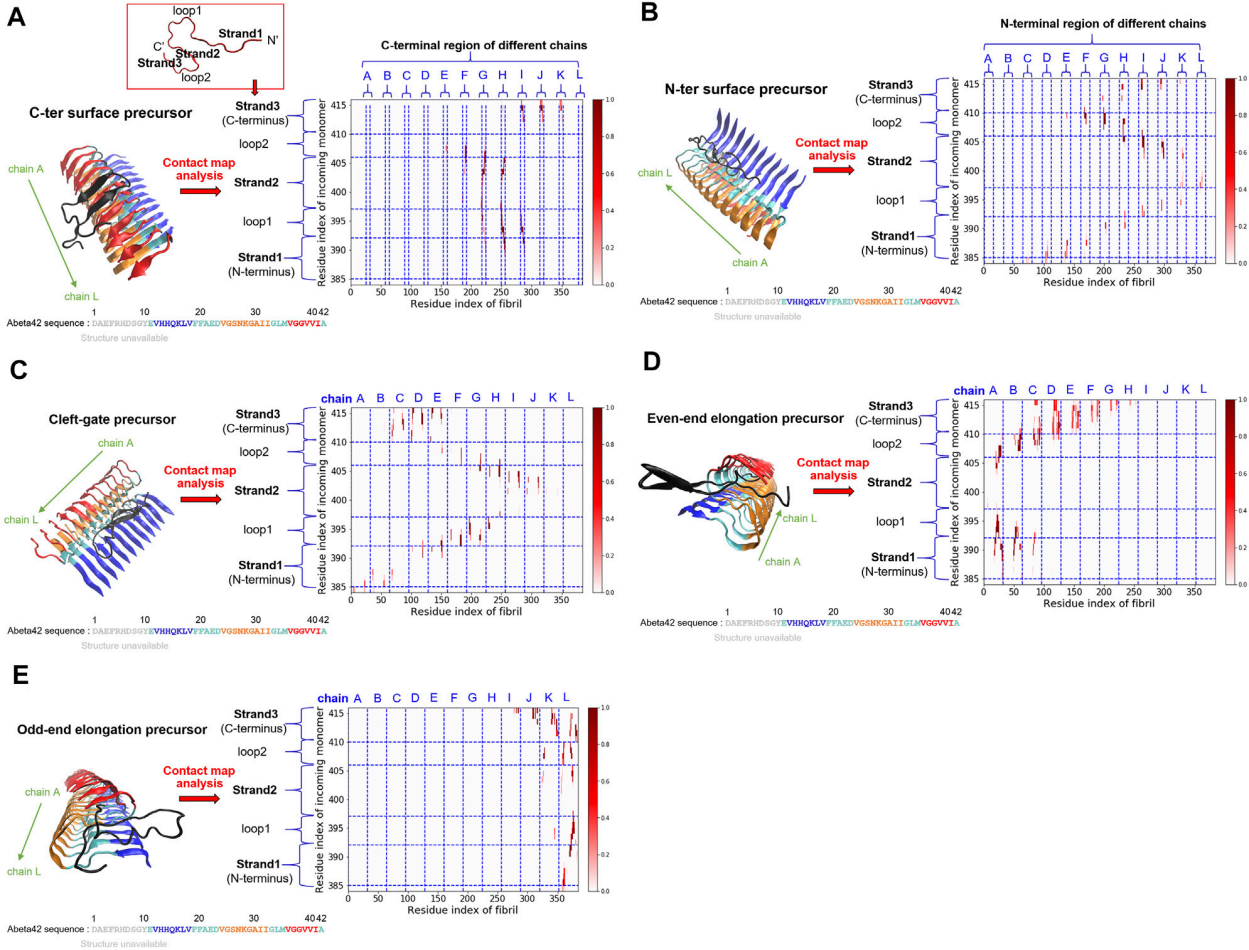
Five key structural precursors on the fibrillar surface and their probability contact maps are shown. **(A)** C-ter surface precursor **(B)** N-ter surface precursor **(C)** Cleft-gate precursor **(D)** Even-end elongation precursor **(E)** Odd-end elongation precursor. The probability contact map next to each structural precursor presents the contacts formed between the monomer and the fibril. The colorbar, scaled by probability, is shown on the right. The horizontal axis uses the fibril index (1–384; 32 × 12 = 384), which sequentially renumbers the 12 monomers in the fibril. The vertical axis describes the residue index of the free monomer by adding up the existing fibril index, 385–416 (384 + 32 = 416). Different structural features are labeled as “strand” or “loop”. Note that in **(A)**, a schematic monomer structure is shown in a red box, with its structure in the fibrillar form. Three strands (strand 1, 2, and 3) and two loops (loop 1 and 2) are indicated. The color scheme for the structure is the same as that used in Figure 1.

From our simulations, we have identified several Abeta binding configurations that potentially can be structural precursors for subsequent surface-dependent processes, e.g., fibril elongation, secondary nucleation. These structural precursors are named with the preferable binding region along the fibrillar surfaces to which the single monomer binds. These binding regions include sites located on the C-terminal (C-ter) surface, the N-terminal (N-ter) surface, the cleft interface, and the two fibril ends (even and odd). Here we would like to characterize their structural features and quantify their contacts with the fibrillar surfaces. The structure of the monomer on the fibrillar surfaces shows primarily a beta-hairpin conformation with their strand vector either parallel (N-ter and cleft-gate precursors) or orthogonal (C-ter, even-end, and odd-end precursors) to the fibril long axis. They are binding configurations of the free energy basin (area III, see Figure 2A). Figure 3 shows the probability contact maps for these structural precursors. The C-ter surface consists of an alignment of hydrophobic sequence segments (36-VGGVVIA-42) along the direction of the fibril axis. Abeta monomer interacts with the C-ter surface primarily through the same VGGVVIA hydrophobic sequence motif of its C-terminus and thus facilitating C-ter/C-ter hydrophobic clustering. In contrast to the C-terminus, the N-ter surface shows a different pattern of contacts. The binding region involves some charged residues in the sequence (11-EVHHQKLVFFAEDVGS-26) along the fibrillar surface. The contact pattern therefore is more diverse, with additional charge-charge interactions (D, E, H and K) that participate in the stabilization of monomer binding. E22 and D23 are particularly important since these two residues are also predicted to be the most highly frustrated areas in the frustration analysis (see below). Figures 3A,B show the C-ter surface contact map and the N-ter surface contact map, respectively.

A somewhat unconventional binding site is identified on the other side of the N-ter surface. Because of its gate-like shape with a cleft at the interface, we name such a monomer binding configuration “cleft-gate” precursor (see Figure 3C). The pattern of the contact map of the cleft-gate precursor looks very similar to the N-ter surface precursor (both display a “S” shape), except that the overall profile is shifted towards the even end. One signature of the cleft-gate precursor is that the contacts formed over the individual chains of the fibril is long-range along the sequence. Therefore, the micro profile of individual chains is different from that of the N-ter surface precursor (see Supplementary Figure S3 in the Supporting Information). Figures 3D,E show the probability contact maps of the even-end and odd-end precursors, respectively. In both cases, the monomer moves to a fibril end and localize onto it. These binding configurations presumably correspond to a fibril being elongating. However, we do not observe the monomer conformation being in the fibril form throughout the simulation. It is very likely that the monomer structural ensemble also plays a role in the process of elongation.

### 3.4 An Elongating Fibril Requires the Added Monomers Being in the “Activated” Conformation

**FIGURE 4:**
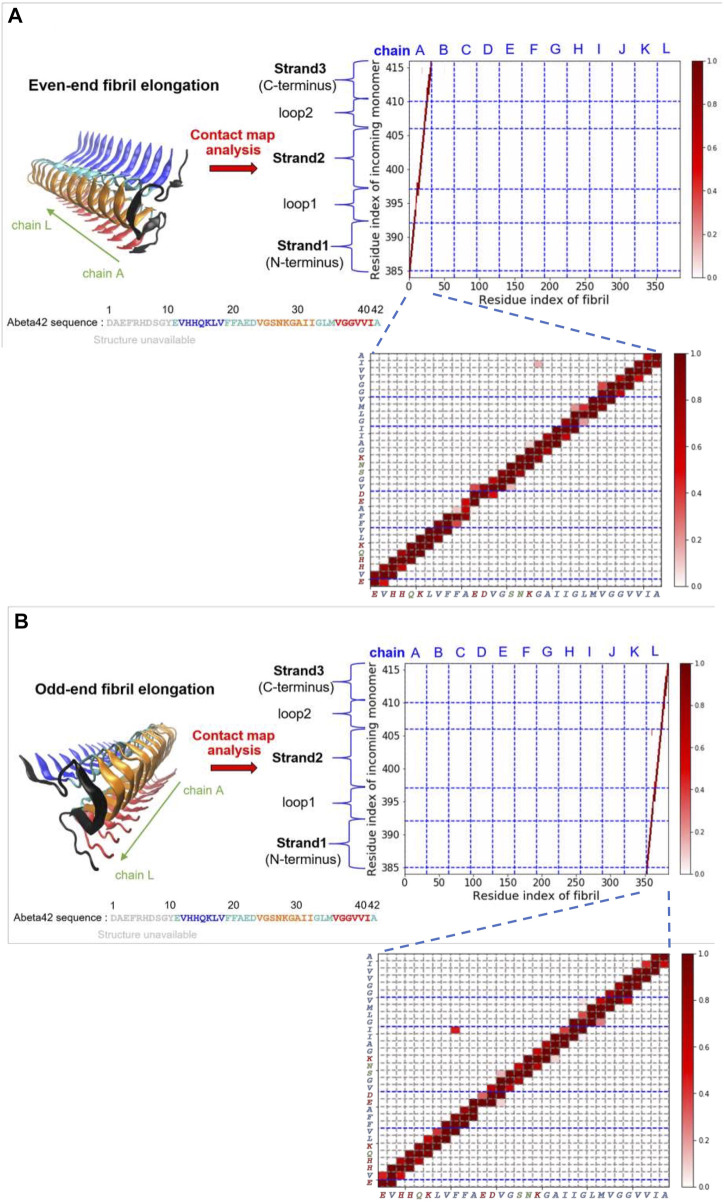
Fibril elongation with the monomer being in the activated fibril-like conformation and their contact maps are shown. **(A)** Even-end fibril elongation. **(B)** Odd-end fibril elongation.

On the other hand, we have also carried out similar probability contact map analysis for simulations using a fibril-like monomer ensemble (“activated” conformation). We find that the structural precursors identified share similar binding sites with those found in the case of relaxed monomer ensemble. This finding implies that some interactions between peptide and fibril on the surface are well conserved, e.g., C-ter/N-ter hydrophobic patches. The overall contact maps of the fibril-like and the relaxed monomer ensembles, however, exhibit quite different features due to the conformational dynamics and rigidity that the monomer intrinsically has. Figure 4 shows the structure of the monomer being elongating on both fibril ends. The simulated structures were taken from the simulation trajectories with the monomer being in the fibril-like conformation. These elongated species, either formed at the even-end (Figure 4A) or the odd-end (Figure 4B), are not observed in the simulations using a structurally unbiased monomer. This result suggests that the monomer conformation being “activated” (conforms to the same shape as in the fibril) plays a determining role in the elongation process while monomer in non-activated form does not significantly contribute to fibril elongation. This result also echoes the two-step dock-and-lock mechanism where the second locking step involves an “activated monomer” that irreversibly binds to the fibril end and elongate (Sasmal et al., 2016). The contact maps for the rest of surface structural precursors can be found in the Supporting Information (Supplementary Figure S7). Supplementary Figures. S4–S7 show the results for the fibril-like monomer binding to the fibrillar surface.

Our simulation study shows that the conformational ensemble of single Abeta plays a key role in determining the kinetic pathways of elongation. The structural rearrangement of the monomer from the “dock” state into the “lock” state involves activated fibril-like conformation that irreversibly binds to the fibril end. This result suggests that the elongation free energy landscape in general can be reduced into a few dimensions: 1. The dimension of the monomer that reversibly searches the landing site over the fibril surface (dock). 2. The dimension of the conformational ensemble of the single monomer on the surface (lock). Once the monomer is in the activated form, the “docking” state merges into the “locking” state. This conversion irreversibly leads to a one-step fibril elongation.

### 3.5 Fibril Surface Binding Site Prediction Using Frustration Analysis

**FIGURE 5:**
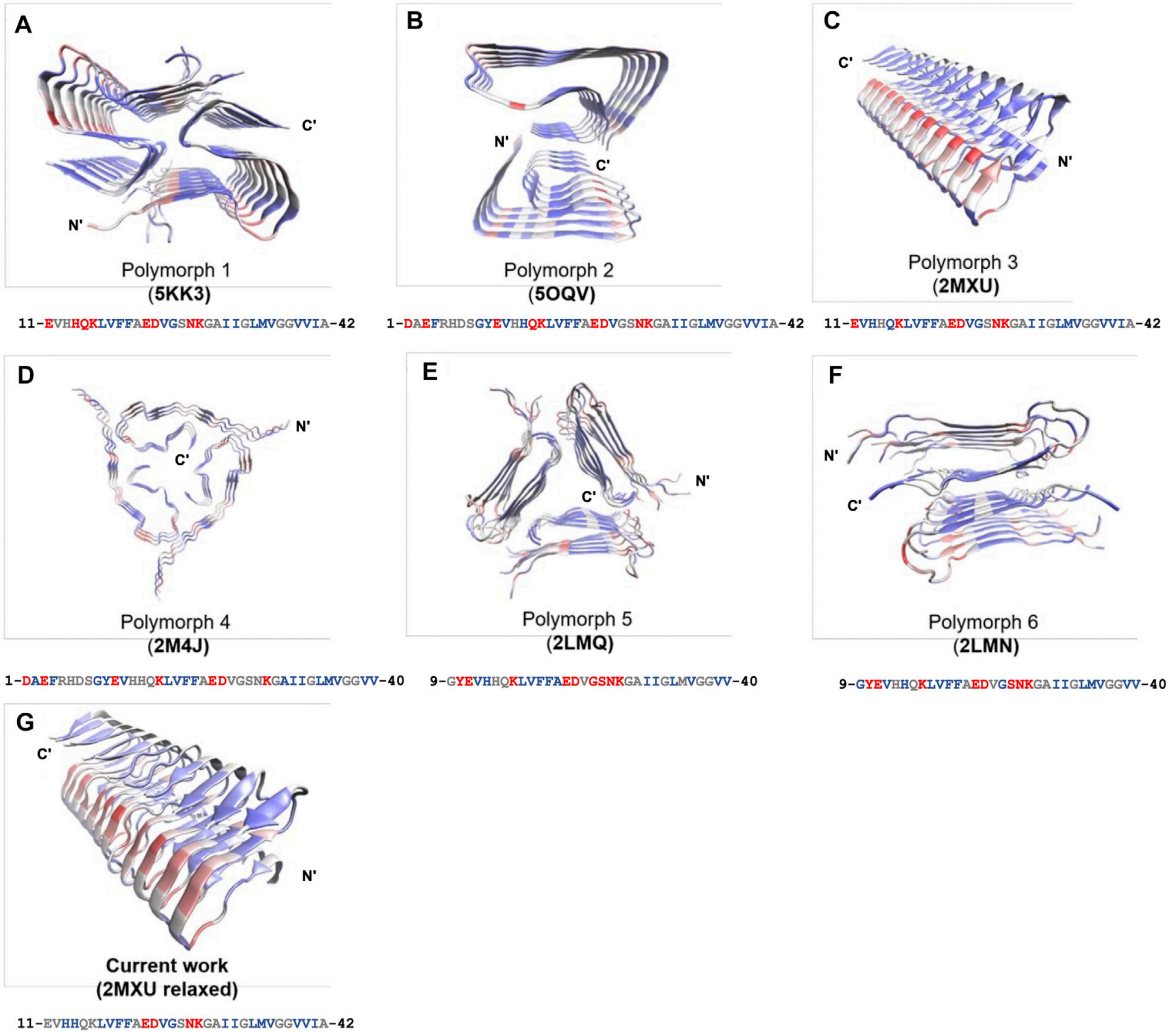
Frustration profiles for a variety of polymorphic fibrillar structures of Abeta peptides available to date. **(A)** 5KK3. **(B)** 5OQV. **(C)** 2MXU. **(D)** 2M4J. **(E)** 2LMQ. **(F)** 2LMN. **(G)** MD-relaxed structure using 2MXU. The magnitude of frustration is quantified using different colors with red (highly frustrated), grey (neutral), and purple (minimally frustrated). The frustration profile is obtained using the frustratometer server (http://frustratometer.qb.fcen.uba.ar/). Single-residue frustration mode is adopted throughout the analysis. Note that for each panel, a 1D sequence representation is shown below (residues are colored using the same frustration color code).

We have shown fibril surface heterogeneity of Abeta protofibril by identifying several binding interfaces. To further our understanding of those predicted sites, we carry out a series of frustration analyses over fibrils of different polymorphs. We aim to compare their results with the results from our MD simulation and provide insight into the predicted sites from an energy perspective. Frustration analysis uses the AWSEM energy functions to access the extent of frustration in the spatially localized interactions in proteins at a residue level (Ferreiro et al., 2007; Parra et al., 2016). If a residue and its neighboring residues are predicted to be highly frustrated, they form a cluster of residues and this cluster may play a role in binding its partners (ex., protein, DNA, RNA, ligands) or serving as an allosteric site. Figure 5 presents the frustration profile for different types of fibrillar polymorphic structures. The highly frustrated residues are shown in red while minimally frustrated residues are shown in purple. We can see that the predicted frustrated areas are not unique but are distributed over the fibrillar surfaces. The exact location of the frustrated area is associated with the structure of the backbone in the given fibrillar architecture. Table 2 summarizes the results of different fibrillar polymorphs. Interestingly, we find that the frustration profiles of different polymorphic structures share several common residues that are predicted to be highly frustrated, although their fibrillar structures are quite different. Charged residues, E22, D23, and K28, for example, are predicted to be highly frustrated across all the fibrillar structures studied. In this work, we also specifically look into the structure of 2MXU. The frustration profile of the 2MXU structure and that of the simulated one are quite similar. This result suggests that structural relaxation due to geometric packing of residues does not significantly affect the frustration profile. For the relaxed fibrillar structure, the most frustrated residues contain E22, D23, N27, K28, which are located in two separate areas in space: (E22, D23) and (N27, K28). The former includes primarily charged residues and is apparently electrostatically driven. The predicted E22-D23 site here agrees well with the binding sites of the N-ter surface, obtained from our MD simulation, to which the monomer binds (see Figure 3B). Here, we have shown an agreement in predicting fibrillar binding sites between the coarse-grained MD simulation and the bioinformatics tool (frustratometer2).

### 3.6 The Helical Twisting Around the Fibril Axis is an Emergent Mechanical Property of a Long Protofilament

**FIGURE 6:**
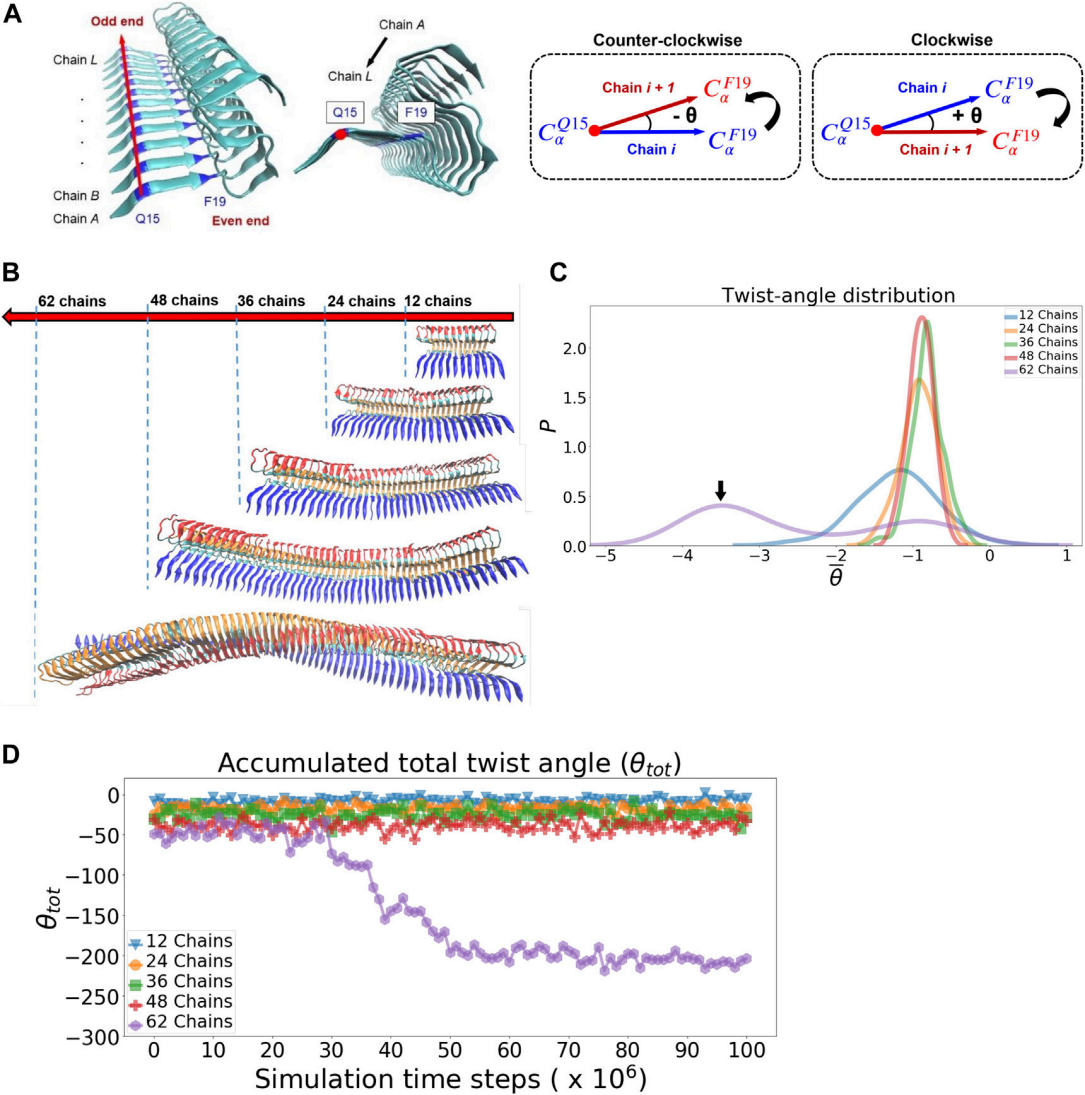
Fibrillar twisting of protofilaments of different sizes is analyzed and compared. **(A)** The definition of the fibrillar twist angle *θ* is shown. **(B)** Representative fibrillar structures of different sizes are displayed (from top, 12, 24, 36, 48, and 62 chains). **(C)** Distribution of the averaged twist angle per chain (*θ̄*) for the protofilaments of different sizes. The black arrow indicates the second peak of the 62-chain protofilament, which represents the twisting structure of the 62-chain protofilament seen in **(B)**. **(D)** Accumulated total twist angle (*θ*_*tot*_) for protofilaments of different sizes. For each of the filament species, the twist angle of each chain is added up to yield a final total twist angle. Note that the initial structure of the protofilament model is taken from the last frame of a pre-equilibration simulation (see Supplementary Figure S10 in the Supporting Information for details).

A variety of protofilament morphologies of antiparallel beta-sheets have been reported (Stroud et al., 2012). In addition to cross-beta structure, one common feature across different fibrillar polymorphs is the helical topology that arises from the degree of overall filament twisting. The overall helical twisting accumulated from the twist angles of individual neighboring pairs of peptide chains. We carry out molecular dynamics simulation for protofilaments of different sizes and examine their twisting features. Starting with the relaxed fibrillar structure that was previously obtained, we used it to build models for the protofilaments with their sizes: 24, 36, 48, up to 62 chains. The calculation of the twist angle along the fibril axis is detailed in Methods, illustrated in Figure 6A. Figure 6B compares the final simulated fibrillar structures of different sizes in the simulation trajectory. The twist angle of individual neighboring chain pairs are recorded in time series. Figure 6C shows the distribution of the averaged twist angle per chain pair *θ̄*. The distribution is primarily a single gaussian-like curve with its peak centered around −1° for protofilaments of size 12 to 48 chains; their corresponding structures are shown in Figure 6B. This averaged twist angle *θ̄* ≈−1° represents the twisted morphology initially obtained from the solid-state NMR structure (2MXU). Interestingly, there exists a small second peak at −3° to −4°, exclusively for the 62-chain protofilament. This small shoulder observed indicates a further filament twisting to a larger degree, as shown in Figure 6B (62-chain protofilament is the last one). The alternative filament twisting observed is consistent with the existing literature on the structure of amyloid fibrils (Bedrood et al., 2012). Figure 6D shows the accumulated total twist angle of protofilament of all sizes as a function of simulation time steps. We find that the alternative twisting feature is not significant for those short filaments. In contrast, *θ̄* = −3° to −4° is not observed until the filament size increases to the number of 62 chains. The result of the 62-chain protofilament clearly shows that the fibrillar structure starts to transform into a left-handed twisted fibrillar form at the time step ≈30 × 10^6^. In other words, filament twisting becomes more significant for protofilaments in a large size. This result suggests that fibrillar twisting is associated with the propagation of localized interactions between neighboring pairs along the fibril axis—*via* cooperative effect. Since the energetics for filament twisting is primarily enthalpy-driven (Periole et al., 2018), all these results support that the filament twisting polymorphic structure is an emergent mechanical property, driven by the size effect of the filament. Such a large-scale mechanical coupling overall contributes to the helical twisting polymorphism of filaments. We show that the simulation protocol used can accurately simulate the mechanical feature of fibrillar twisting as well as the global structural rearrangement of amyloid protofilaments.

## 4 Conclusion

The interaction of a free amyloid protein monomer with a pre-existing fibrillar surface is an essential process which initiates subsequent fibril growth. Efficient fibril growth is mediated by several secondary processes such as elongation and surface-catalyzed nucleation. Understanding their kinetic pathways can provide mechanistic insight into the molecular mechanism of fibril growth. In this study, we have constructed a simulation platform for studying the early stage of fibril growth using a new GPU-enabled coarse-grained protein force field (openAWSEM). This simulation platform allows us to carry out long time simulations for protein aggregation over a fibrillar surface. We have investigated the thermodynamic binding affinity for a single monomer binding to fibrillar surfaces and find out that surface heterogeneity can significantly influence the predicted binding affinity. Accordingly, we have also identified several key surface binding sites: C-ter, N-ter, cleft, even-end, odd-end. Our study reveals several monomer-fibril binding configurations which potentially are amyloid precursors for subsequent elongation and secondary nucleation. This finding suggests that surface heterogeneity, entailed by the protein sequence and the resulting self-assembly, plays a key role in determining the aggregation pathways and, more importantly, it inevitably leads to variation in the thermodynamic binding affinity. In addition, we have used a bioinformatics tool to predict binding sites over different polymorphic fibrillar surfaces. For the fibril structure of interest, the frustration analysis predicts several potential functional sites, including residue E22 and D23 (mostly frustrated). These residues belong to the N-ter surface identified from the simulation. This binding site presumably can be modulated electrostatically (e.g., pH, ionic strength) to reflect its binding plasticity. To understand surface properties of fibrils in response to the global fibrillar twisting, we have simulated fibrillar twisting of single protofilaments with different sizes. Our result shows that fibrillar twisting is an emergent, collective property that correlates with the number of monomers participating in the fibril. We propose that the length-dependent fibrillar twisting may influence the population distribution of the amyloid precursors and thus drive different aggregation pathways for fibril growth. This work demonstrates the capability of the current simulation protocol for a comprehensive survey over fibril stability, binding affinity, surface heterogeneity, and mechanical twisting of polymorphic protofilaments. All these properties are prerequisite for understanding the molecular mechanism of surface-catalyzed secondary processes. We leave that task for future work.

## Data Availability

The datasets (generated/ANALYZED) for this study can be found in the code repository of MYTLab/pyAggreg on Github. (https://github.com/MYTLab/pyAggreg).

## References

[B1] Bedrood S. Li Y. Isas J. M. Hegde B. G. Baxa U. Haworth I. S. (2012). Fibril Structure of Human Islet Amyloid Polypeptide. J. Biol. Chem. 287, 5235–5241. 10.1074/jbc.m111.327817 22187437 PMC3285303

[B2] Cecchini M. Rao F. Seeber M. Caflisch A. (2004). Replica Exchange Molecular Dynamics Simulations of Amyloid Peptide Aggregation. J. Chem. Phys. 121, 10748–10756. 10.1063/1.1809588 15549960

[B3] Charest N. Tro M. Bowers M. T. Shea J.-E. (2020). Latent Models of Molecular Dynamics Data: Automatic Order Parameter Generation for Peptide Fibrillization. J. Phys. Chem. B 124, 8012–8022. 10.1021/acs.jpcb.0c05763 32790375

[B4] Chen M. Chen X. Schafer N. P. Clementi C. Komives E. A. Ferreiro D. U. (2020). Surveying Biomolecular Frustration at Atomic Resolution. Nat. Commun. 11, 5944. 10.1038/s41467-020-19560-9 33230150 PMC7683549

[B5] Chen M. Lin X. Lu W. Schafer N. P. Onuchic J. N. Wolynes P. G. (2018a). Template-Guided Protein Structure Prediction and Refinement Using Optimized Folding Landscape Force fields. J. Chem. Theor. Comput. 14, 6102–6116. 10.1021/acs.jctc.8b00683 PMC6713208 30240202

[B6] Chen M. Schafer N. P. Wolynes P. G. (2018b). Surveying the Energy Landscapes of Aβ Fibril Polymorphism Fibril Polymorphism. J. Phys. Chem. B 122, 11414–11430. 10.1021/acs.jpcb.8b07364 30215519 PMC6713213

[B7] Chen M. Tsai M. Zheng W. Wolynes P. G. (2016). The Aggregation Free Energy Landscapes of Polyglutamine Repeats. J. Am. Chem. Soc. 138, 15197–15203. 10.1021/jacs.6b08665 27786478 PMC5803750

[B8] Chen X.-Q. Mobley W. C. (2019). Alzheimer Disease Pathogenesis: Insights from Molecular and Cellular Biology Studies of Oligomeric Aβ and Tau Species and Tau Species. Front. Neurosci. 13, 659. 10.3389/fnins.2019.00659 31293377 PMC6598402

[B9] Cohen S. I. A. Cukalevski R. Michaels T. C. T. Šarić A. Törnquist M. Vendruscolo M. (2018). Distinct Thermodynamic Signatures of Oligomer Generation in the Aggregation of the Amyloid-β Peptide Peptide. Nat. Chem. 10, 523–531. 10.1038/s41557-018-0023-x 29581486 PMC5911155

[B10] Colvin M. T. Silvers R. Ni Q. Z. Can T. V. Sergeyev I. Rosay M. (2016). Atomic Resolution Structure of Monomorphic Aβ42 Amyloid Fibrils42 Amyloid Fibrils. J. Am. Chem. Soc. 138, 9663–9674. 10.1021/jacs.6b05129 27355699 PMC5389415

[B11] Crespo R. Rocha F. A. Damas A. M. Martins P. M. (2012). A Generic Crystallization-like Model that Describes the Kinetics of Amyloid Fibril Formation. J. Biol. Chem. 287, 30585–30594. 10.1074/jbc.m112.375345 22767606 PMC3436372

[B12] Daskalov A. Martinez D. Coustou V. El Mammeri N. Berbon M. Andreas L. B. (2021). Structural and Molecular Basis of Cross-Seeding Barriers in Amyloids. Proc. Natl. Acad. Sci. U. S. A. 118, e2014085118. 10.1073/pnas.2014085118 33443172 PMC7817211

[B13] Davtyan A. Schafer N. P. Zheng W. Clementi C. Wolynes P. G. Papoian G. A. (2012). AWSEM-MD: Protein Structure Prediction Using Coarse-Grained Physical Potentials and Bioinformatically Based Local Structure Biasing. J. Phys. Chem. B. 116, 8494–8503. 10.1021/jp212541y 22545654 PMC3406225

[B14] Deng Y. Roux B. (2009). Computations of Standard Binding Free Energies with Molecular Dynamics Simulations. J. Phys. Chem. B. 113, 2234–2246. 10.1021/jp807701h 19146384 PMC3837708

[B15] Di Palma F. Bottaro S. Bussi G. (2015). Kissing Loop Interaction in Adenine Riboswitch: Insights from Umbrella Sampling Simulations. BMC Bioinformatics. 16 (Suppl. 9), S6. 10.1186/1471-2105-16-S9-S6 PMC4464220 26051557

[B16] [Dataset] Eastman P. Swails J. Chodera J. D. McGibbon R. T. Zhao Y. Beauchamp K. A. (2017). OpenMM 7: Rapid Development of High Performance Algorithms for Molecular Dynamics. Plos Comput. Biol. 13, e1005659. 10.1371/journal.pcbi.1005659 28746339 PMC5549999

[B17] Fändrich M. Nyström S. Nilsson K. P. R. Böckmann A. LeVine H. 3rd Hammarström P. (2018). Amyloid Fibril Polymorphism: a challenge for Molecular Imaging and Therapy. J. Intern. Med. 283, 218–237. 10.1111/joim.12732 29360284 PMC5820168

[B18] Ferreiro D. U. Hegler J. A. Komives E. A. Wolynes P. G. (2007). Localizing Frustration in Native Proteins and Protein Assemblies. Proc. Natl. Acad. Sci. 104, 19819–19824. 10.1073/pnas.0709915104 18077414 PMC2148382

[B19] Ferreiro D. U. Komives E. A. Wolynes P. G. (2018). Frustration, Function and Folding. Curr. Opin. Struct. Biol. 48, 68–73. 10.1016/j.sbi.2017.09.006 29101782 PMC6005193

[B20] Grazioli G. Martin R. W. Butts C. T. (2019). Comparative Exploratory Analysis of Intrinsically Disordered Protein Dynamics Using Machine Learning and Network Analytic Methods. Front. Mol. Biosci. 6, 42. 10.3389/fmolb.2019.00042 31245383 PMC6581705

[B21] Gremer L. Schölzel D. Schenk C. Reinartz E. Labahn J. Ravelli R. B. G. (2017). Fibril Structure of Amyloid-Β(1-42) by Cryo-Electron Microscopy(1-42) by Cryo-Electron Microscopy. Science 358, 116–119. 10.1126/science.aao2825 28882996 PMC6080689

[B22] Han W. Schulten K. (2014). Fibril Elongation by Aβ17-42: Kinetic Network Analysis of Hybrid-Resolution Molecular Dynamics Simulations17–42: Kinetic Network Analysis of Hybrid-Resolution Molecular Dynamics Simulations. J. Am. Chem. Soc. 136, 12450–12460. 10.1021/ja507002p 25134066 PMC4156860

[B23] Humphrey W. Dalke A. Schulten K. (1996). VMD: Visual Molecular Dynamics. J. Mol. Graph. 14, 33–38. 10.1016/0263-7855(96)00018-5 8744570

[B24] Ilie I. M. Caflisch A. (2018). Disorder at the Tips of a Disease-Relevant Aβ42 Amyloid Fibril: A Molecular Dynamics Study42 Amyloid Fibril: A Molecular Dynamics Study. J. Phys. Chem. B 122, 11072–11082. 10.1021/acs.jpcb.8b05236 29965774

[B25] Jin S. Contessoto V. G. Chen M. Schafer N. P. Lu W. Chen X. (2020). AWSEM-suite: a Protein Structure Prediction Server Based on Template-Guided, Coevolutionary-Enhanced Optimized Folding Landscapes. Nucleic Acids Res. 48, W25–W30. 10.1093/nar/gkaa356 32383764 PMC7319565

[B26] Kollmer M. Close W. Funk L. Rasmussen J. Bsoul A. Schierhorn A. (2019). Cryo-EM Structure and Polymorphism of Aβ Amyloid Fibrils Purified from Alzheimer's Brain Tissue Amyloid Fibrils Purified from Alzheimer’s Brain Tissue. Nat. Commun. 10, 4760. 10.1038/s41467-019-12683-8 31664019 PMC6820800

[B27] Konno H. Watanabe-Nakayama T. Uchihashi T. Okuda M. Zhu L. Kodera N. (2020). Dynamics of Oligomer and Amyloid Fibril Formation by Yeast Prion Sup35 Observed by High-Speed Atomic Force Microscopy. Proc. Natl. Acad. Sci. USA 117, 7831–7836. 10.1073/pnas.1916452117 32213585 PMC7149427

[B28] [Dataset] Kumar S. Rosenberg J. M. Bouzida D. Swendsen R. H. Kollman P. A. (1992). THE Weighted Histogram Analysis Method for Free-Energy Calculations on Biomolecules. I. The Method. J. Comput. Chem. 13, 1011–1021. 10.1002/jcc.540130812

[B29] Laughlin R. B. Pines D. Schmalian J. Stojkovic B. P. Wolynes P. (2000). The Middle Way. Proc. Natl. Acad. Sci. U S A. 97, 32–37. 10.1073/pnas.97.1.32 10618366 PMC26611

[B30] Lu W. Bueno C. Schafer N. P. Moller J. Jin S. Chen M. (2020). OpenAWSEM with Open3SPN2: a Fast, Flexible, and Accessible Framework for Large-Scale Coarse-Grained Biomolecular Simulations. Plos Comput. Biol. 17, e1008308. 10.1371/journal.pcbi.1008308 PMC7906472 33577557

[B31] [Dataset] Marcovitz A. Levy Y. (2013). Weak Frustration Regulates Sliding and Binding Kinetics on Rugged Protein–DNA Landscapes. J. Phys. Chem. B. 117, 13005–13014. 10.1021/jp402296d 23668488

[B32] McGibbon R. T. Beauchamp K. A. Harrigan M. P. Klein C. Swails J. M. Hernández C. X. (2015). MDTraj: A Modern Open Library for the Analysis of Molecular Dynamics Trajectories. Biophysical J. 109, 1528–1532. 10.1016/j.bpj.2015.08.015 PMC4623899 26488642

[B33] Okumura H. Itoh S. G. (2016). Structural and Fluctuational Difference between Two Ends of Aβ Amyloid Fibril: MD Simulations Predict Only One End Has Open Conformations Amyloid Fibril: MD Simulations Predict Only One End Has Open Conformations. Sci. Rep. 6, 38422. 10.1038/srep38422 27934893 PMC5146922

[B34] [Dataset] O’Nuallain B. Shivaprasad S. Kheterpal I. Wetzel R. (2005). Thermodynamics of A*β*(1–40) Amyloid Fibril Elongation. Biochemistry 44, 12709–12718. 10.1021/bi050927h 16171385

[B35] Parra R. G. Schafer N. P. Radusky L. G. Tsai M.-Y. Guzovsky A. B. Wolynes P. G. (2016). Protein Frustratometer 2: a Tool to Localize Energetic Frustration in Protein Molecules, Now with Electrostatics. Nucleic Acids Res. 44, W356–W360. 10.1093/nar/gkw304 27131359 PMC4987889

[B36] Periole X. Huber T. Bonito-Oliva A. Aberg K. C. van der Wel P. C. A. Sakmar T. P. (2018). Energetics Underlying Twist Polymorphisms in Amyloid Fibrils. J. Phys. Chem. B 122, 1081–1091. 10.1021/acs.jpcb.7b10233 29254334 PMC5857390

[B37] Potoyan D. A. Bueno C. Zheng W. Komives E. A. Wolynes P. G. (2017). Resolving the NFκB Heterodimer Binding Paradox: Strain and Frustration Guide the Binding of Dimeric Transcription FactorsB Heterodimer Binding Paradox: Strain and Frustration Guide the Binding of Dimeric Transcription Factors. J. Am. Chem. Soc. 139, 18558–18566. 10.1021/jacs.7b08741 29183131 PMC5803749

[B38] Potoyan D. A. Zheng W. Ferreiro D. U. Wolynes P. G. Komives E. A. (2016a). PEST Control of Molecular Stripping of NFκB from DNA Transcription SitesB from DNA Transcription Sites. J. Phys. Chem. B 120, 8532–8538. 10.1021/acs.jpcb.6b02359 27098223 PMC5389414

[B39] Potoyan D. A. Zheng W. Komives E. A. Wolynes P. G. (2016b). Molecular Stripping in theNF-κB/IκB/DNAgenetic Regulatory networkB/IB/DNA Genetic Regulatory Network. Proc. Natl. Acad. Sci. USA 113, 110–115. 10.1073/pnas.1520483112 26699500 PMC4711861

[B40] Qiang W. Kelley K. Tycko R. (2013). Polymorph-Specific Kinetics and Thermodynamics of β-Amyloid Fibril Growth-Amyloid Fibril Growth. J. Am. Chem. Soc. 135, 6860–6871. 10.1021/ja311963f 23627695 PMC3686096

[B41] Riek R. Eisenberg D. S. (2016). The Activities of Amyloids from a Structural Perspective. Nature 539, 227–235. 10.1038/nature20416 27830791

[B42] Rodriguez R. A. Chen L. Y. Plascencia-Villa G. Perry G. (2018). Thermodynamics of Amyloid-β Fibril Elongation: Atomistic Details of the Transition State Fibril Elongation: Atomistic Details of the Transition State. ACS Chem. Neurosci. 9, 783–789. 10.1021/acschemneuro.7b00409 29239603 PMC5911799

[B43] Sasmal S. Schwierz N. Head-Gordon T. (2016). Mechanism of Nucleation and Growth of Aβ40 Fibrils from All-Atom and Coarse-Grained Simulations40 Fibrils from All-Atom and Coarse-Grained Simulations. J. Phys. Chem. B 120, 12088–12097. 10.1021/acs.jpcb.6b09655 27806205

[B44] Scheidt T. Łapińska U. Kumita J. R. Whiten D. R. Klenerman D. Wilson M. R. (2019). Secondary Nucleation and Elongation Occur at Different Sites on Alzheimer’s Amyloid- Aggregates. Sci. Adv. 5, eaau3112. 10.1126/sciadv.aau3112 31001578 PMC6469941

[B45] Scherer M. K. Trendelkamp-Schroer B. Paul F. Pérez-Hernández G. Hoffmann M. Plattner N. (2015). PyEMMA 2: A Software Package for Estimation, Validation, and Analysis of Markov Models. J. Chem. Theor. Comput. 11, 5525–5542. 10.1021/acs.jctc.5b00743 26574340

[B46] [Dataset] Schmidt M. Sachse C. Richter W. Xu C. Fandrich M. Grigorieff N.. (2009). Comparison of Alzheimer a (1-40) and a (1-42) Amyloid Fibrils Reveals Similar Protofilament Structures 10.1073/pnas.0905007106 PMC2764733 19843697

[B47] Schwierz N. Frost C. V. Geissler P. L. Zacharias M. (2016). Dynamics of Seeded Aβ40-Fibril Growth from Atomistic Molecular Dynamics Simulations: Kinetic Trapping and Reduced Water Mobility in the Locking Step40-Fibril Growth from Atomistic Molecular Dynamics Simulations: Kinetic Trapping and Reduced Water Mobility in the Locking Step. J. Am. Chem. Soc. 138, 527–539. 10.1021/jacs.5b08717 26694883

[B48] Schwierz N. Frost C. V. Geissler P. L. Zacharias M. (2017). From Aβ Filament to Fibril: Molecular Mechanism of Surface-Activated Secondary Nucleation from All-Atom MD Simulations Filament to Fibril: Molecular Mechanism of Surface-Activated Secondary Nucleation from All-Atom MD Simulations. J. Phys. Chem. B 121, 671–682. 10.1021/acs.jpcb.6b10189 27992231

[B49] Sirovetz B. J. Schafer N. P. Wolynes P. G. (2017). Protein Structure Prediction: Making AWSEM AWSEM‐ER by Adding Evolutionary Restraints. Proteins 85, 2127–2142. 10.1002/prot.25367 28799172 PMC5807005

[B50] Stroud J. C. Liu C. Teng P. K. Eisenberg D. (2012). Toxic Fibrillar Oligomers of Amyloid- Have Cross- Structure Have Cross- Structure. Proc. Natl. Acad. Sci. 109, 7717–7722. 10.1073/pnas.1203193109 22547798 PMC3356606

[B51] Thacker D. Sanagavarapu K. Frohm B. Meisl G. Knowles T. P. J. Linse S. (2020). The Role of Fibril Structure and Surface Hydrophobicity in Secondary Nucleation of Amyloid Fibrils. Proc. Natl. Acad. Sci. USA 117, 25272–25283. 10.1073/pnas.2002956117 33004626 PMC7568274

[B52] Tsai M.-Y. (2019). Role of Physical Nucleation Theory in Understanding Conformational Conversion between Pathogenic and Nonpathogenic Aggregates of Low-Complexity Amyloid Peptides. Res. Chem. Intermed. 45, 5357–5373. 10.1007/s11164-019-03974-2

[B53] Tsai M.-Y. Yuan J.-M. Lin S.-H. (2015). Thermodynamic Insight into Protein Aggregation Using a Kinetic Ising Model. Jnl Chin. Chem. Soc 62, 21–25. 10.1002/jccs.201400272

[B54] Tsai M.-Y. Zhang B. Zheng W. Wolynes P. G. (2016a). Molecular Mechanism of Facilitated Dissociation of Fis Protein from DNA. J. Am. Chem. Soc. 138, 13497–13500. 10.1021/jacs.6b08416 27685351 PMC5805664

[B55] Tsai M.-Y. Zheng W. Balamurugan D. Schafer N. P. Kim B. L. Cheung M. S. (2016b). Electrostatics, Structure Prediction, and the Energy Landscapes for Protein Folding and Binding. Protein Sci. 25, 255–269. 10.1002/pro.2751 26183799 PMC4815325

[B56] Tsai M.-Y. Zheng W. Chen M. Wolynes P. G. (2019). Multiple Binding Configurations of Fis Protein Pairs on DNA: Facilitated Dissociation versus Cooperative Dissociation. J. Am. Chem. Soc. 141, 18113–18126. 10.1021/jacs.9b08287 31566963

[B57] Tycko R. (2015). Amyloid Polymorphism: Structural Basis and Neurobiological Relevance. Neuron 86, 632–645. 10.1016/j.neuron.2015.03.017 25950632 PMC4425266

[B58] Wälti M. A. Ravotti F. Arai H. Glabe C. G. Wall J. S. Böckmann A. (2016). Atomic-resolution Structure of a Disease-Relevant Aβ(1-42) Amyloid Fibril(1-42) Amyloid Fibril. Proc. Natl. Acad. Sci. USA 113, E4976–E4984. 10.1073/pnas.1600749113 27469165 PMC5003276

[B59] Xiao Y. Ma B. McElheny D. Parthasarathy S. Long F. Hoshi M. (2015). Aβ(1-42) Fibril Structure Illuminates Self-Recognition and Replication of Amyloid in Alzheimer's Disease. Nat. Struct. Mol. Biol. 22, 499–505. 10.1038/nsmb.2991 25938662 PMC4476499

[B60] Xu Y. Safari M. S. Ma W. Schafer N. P. Wolynes P. G. Vekilov P. G. (2019). Steady, Symmetric, and Reversible Growth and Dissolution of Individual Amyloid-β Fibrils Fibrils. ACS Chem. Neurosci. 10, 2967–2976. 10.1021/acschemneuro.9b00179 31099555

[B61] Xue W.-F. Homans S. W. Radford S. E. (2008). Systematic Analysis of Nucleation-dependent Polymerization Reveals New Insights into the Mechanism of Amyloid Self-Assembly. Pnas 105, 8926–8931. 10.1073/pnas.0711664105 18579777 PMC2440360

[B62] Zhang B. Zheng W. Papoian G. A. Wolynes P. G. (2016). Exploring the Free Energy Landscape of Nucleosomes. J. Am. Chem. Soc. 138, 8126–8133. 10.1021/jacs.6b02893 27300314

[B63] Zhang R. Hu X. Khant H. Ludtke S. J. Chiu W. Schmid M. F. (2009). Interprotofilament Interactions between Alzheimer's A 1-42 Peptides in Amyloid Fibrils Revealed by cryoEM. Proc. Natl. Acad. Sci. 106, 4653–4658. 10.1073/pnas.0901085106 19264960 PMC2660777

[B64] Zheng W. Schafer N. P. Davtyan A. Papoian G. A. Wolynes P. G. (2012). Predictive Energy Landscapes for Protein-Protein Association. Proc. Natl. Acad. Sci. 109, 19244–19249. 10.1073/pnas.1216215109 23129648 PMC3511104

[B65] Zheng W. Tsai M.-Y. Chen M. Wolynes P. G. (2016). Exploring the Aggregation Free Energy Landscape of the Amyloid-β Protein (1-40) Protein (1-40). Proc. Natl. Acad. Sci. USA 113, 11835–11840. 10.1073/pnas.1612362113 27698130 PMC5081630

[B66] Zheng W. Tsai M.-Y. Wolynes P. G. (2017). Comparing the Aggregation Free Energy Landscapes of Amyloid Beta(1-42) and Amyloid Beta(1-40). J. Am. Chem. Soc. 139, 16666–16676. Accepted. 10.1021/jacs.7b08089 29057654 PMC5805378

