# Corrigendum: Fibril Surface-Dependent Amyloid Precursors Revealed by Coarse-Grained Molecular Dynamics Simulation

**DOI:** 10.3389/fmolb.2022.944884

**Published:** 2022-06-20

**Authors:** Yuan-Wei Ma, Tong-You Lin, Min-Yeh Tsai

**Affiliations:** Department of Chemistry, Tamkang University, New Taipei City, Taiwan

**Keywords:** abeta, MD simulation, coarse-grained model, fibril surface, secondary nucleation, fibrillar twisting, binding sites, elongation (growth)

In the original article, there was a mistake in Figure 2 and Table 1 as published (Supplementary Figures S1, S2, and S4–S6 in the Supplementary Material are also affected). The mistake was made by using the wrong residue index (it should have been N27, but S26 was erroneously used instead) in the WHAM analysis for generating a free energy profile. The corrected Figure 2 and Table 1 are shown below. The updated supplementary figures are available in the Supplementary Material.

**FIGURE 2:**
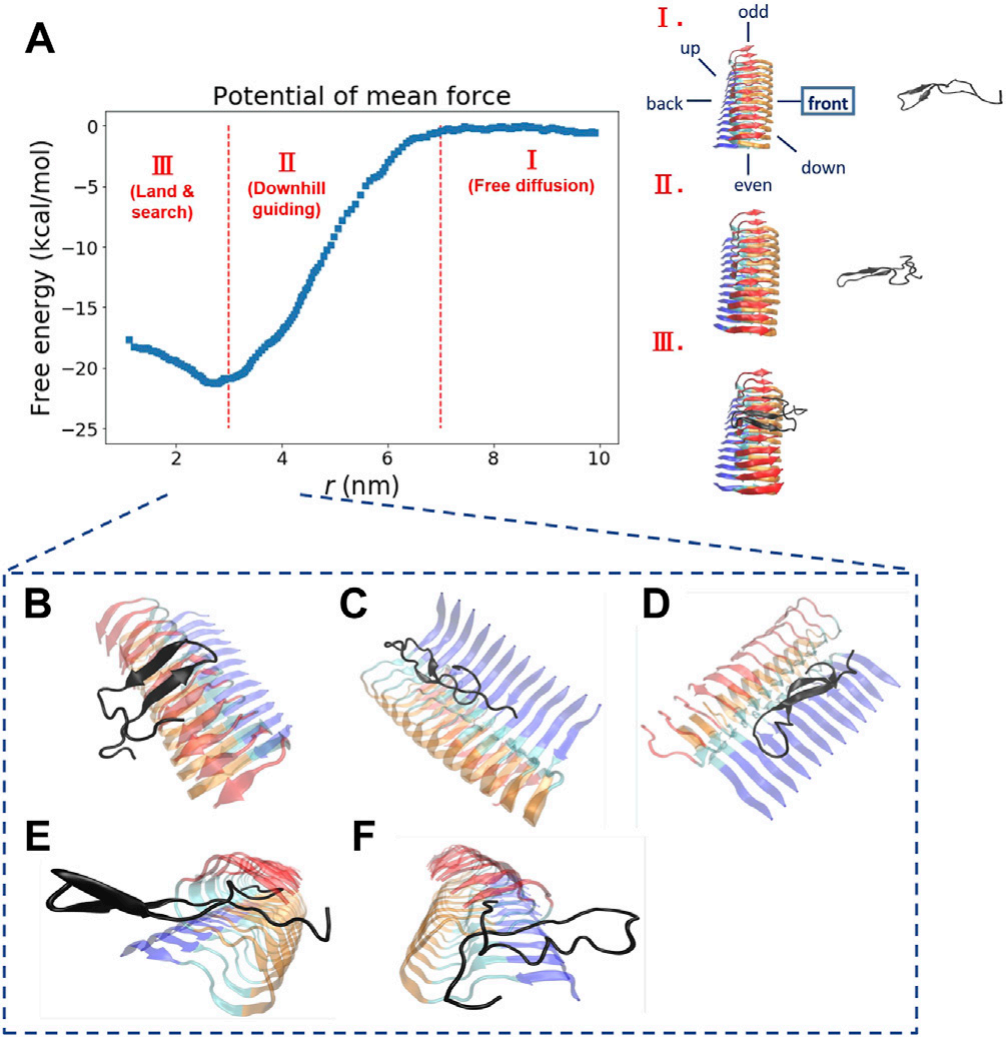
The free energy profile for a single Abeta11-42 monomer binding to the Abeta42 fibrillar surface (12 chains) is shown. **(A)** The free energy profile features three different aggregation stages, labeled as I. Free diffusion. II. Downhill guiding. III. Dock and lock. r is defined as the distance between the C-beta of residue 27th in the free monomer and the C-beta of residue 27th of chain F in the fibril. A representative configuration at each aggregation stage is schematically shown on the right. Simulations were prepared with six different monomer positions with respect to the central pre-existing fibril. Six orientations: front, back, up, down, even, odd are schematically shown in the diagram. The free energy profile shown refers to the result obtained from the simulation setup with the monomer positioned in the “front” orientation. The free energy profiles for the rest of the orientations are shown in the Supporting Information (Supplementary Figure S2). **(B–F)** The representative structures taken from stage III in which different binding configurations are formed upon the monomer landing and searching over the fibrillar surface. These binding configurations are potentially surface-catalyzed precursors for fibril growth. **(B)** C-ter surface precursor **(C)** N-ter surface precursor **(D)** Cleft-gate precursor **(E)** Even-end precursor **(F)** Odd-end precursor.

**TABLE 1:** Thermodynamic binding affinity of Abeta binding to a fibril.

∆Gb (kcal/mol)	Top	Length	Solubility (µM)	Condition	Type
−8.7	Twofold	1–40	0.44	<10 µM (27°C)	Exp. Xu et al. (2019)
−8.7	Twofold	1–40	0.3–0.4	<75 µM (24°C)	Exp. Qiang et al. (2013)
−9	—	1–40	0.8–1.0	<30 µM (37°C)	Exp. O’Nuallain et al. (2005)
−12 (even)	Twofold	17–42	—	37°C	Sim. Han and Schultan, (2014)
−11.3 (odd)					
−15.6	Twofold	9–40	—	37°C	Sim. Schwiers et al. (2017)
−25.8	Single	11–42	—	27°C	This Work

In the original article, there was an error. A wrong distance value was used, and several wrong free energy values were used.

A correction has been made to **3 Results and Discussion, 3.2 A Monomer Binding to Fibrillar Surfaces can be Characterized** … , paragraphs 1 and 2. The corrected paragraph excerpts appear below:

“We explore the free energy landscape along a distance separation between a free Abeta monomer and a fibril surface. To enhance sampling over different spatial orientations, we carry out several independent simulations with different initial positions of the monomer with respect to the central fibril. A total of six different positions were chosen to address the fluctuations of orientation. The six simulations, having the monomer being put in different orientations: front, back, up, down, even, and odd, respectively, were performed (see the subplot in Figure 2A for a schematic description). Figure 2A presents a representative free energy profile with the monomer being positioned in the “front” position. From the free energy profile, several features can be observed. They are classified into three different stages accordingly: I. Free diffusion. II. Downhill guiding. III. Dock and lock. When the Abeta monomer is far from the central fibril (*r* > 80 Å), the dynamics of the free monomer is primarily diffusive and that the free energy profile is nearly flat in the plateau (Stage I). As the distance between the fibril and the monomer decreases, the monomer is subject to a long-range guiding force due to electrostatics, and therefore, the monomer begins to approach the fibril. This long-range guidance yields an energetically downhill profile (Stage II). The downhill free energy continues until its slope significantly changes at *r* ≈ 30 Å where the free energy profile displays a curvature. After that, the monomer begins to have physical contacts with the fibril (Stage III). In stage III, there are many ways for the monomer to dock the fibril. The biasing strategy used allows spatially orientational flexibility for the monomer to dock the fibril. As a result, the monomer is able to dock the fibrillar surface through different sites. All the resulting binding configurations lead to a clear free energy basin at *r* ≈ 30 Å. … .”

“Next, we look into the thermodynamic binding affinity, defined by the potential of mean force (PMF). In determining the free energy of binding, multiple free energy calculations have shown variation in *r*_*b*_ (*r*_*b*_ refers to the distance at which the global free energy basin is found), suggesting that the Abeta monomer binds to the fibril surface through a pathway-dependent manner. This pathway dependence very likely causes some variations in the binding free energy profiles since the monomer might interact with the fibril surfaces through different “dock” sites. Here, we do not assume any specific binding site a priori for the monomer to bind with. Instead, we aim to sample different binding trajectories and then combine these trajectories to determine the standard binding affinity (with c_0_ 1 M, see Section 2.2.1). The value is computed to be −25.8 ± 2.4 kcal/mol if we use the data of all the six orientations to ensure the orientational fluctuations. The binding affinity, determined by the simulation trajectory of individual single orientation, ranges from −23 to −29 kcal/mol. …”

The authors apologize for these errors and state that this does not change the scientific conclusions of the article in any way. The original article has been updated.

