# Supplementary material for: Fibril Surface-Dependent Amyloid Precursors Revealed by Coarse-Grained Molecular Dynamics Simulation
Source: Front Mol Biosci. 2021 Aug 6;8:719320. doi: 10.3389/fmolb.2021.719320 (PMC8378332; doi:10.3389/fmolb.2021.719320)
Supplement: Supplementary file 1 [file DataSheet1.pdf]

Supporting Information

Fibril Surface-dependent Amyloid Precursors revealed by Coarse-grained Molecular Dynamics Simulation

Yuan-Wei Ma, Tong-You Lin, and Min-Yeh Tsai*

Department of Chemistry, Tamkang University, New Taipei City, Taiwan (R.O.C.) 251301

April 7, 2022

Figure S1. Distribution of sampling windows for simulation with Abeta11-42 peptide binding to the fibrillar surface is shown. **2**

Figure S2. The free energy profile for an Abeta11-42 monomer (unbiased) binding to the fibril. **3**

Figure S3. Probability contact maps for the five different structural precursors on the fibrillar surface are shown to a residue-to-residue resolution. **5**

Figure S4. The free energy profile for a single fibril-like ensemble Abeta11-42 monomer binding to the Abeta42 fibrillar surface (12 chains) is shown (monomer in the ”back” orientation). **6**

Figure S5. Distribution of sampling windows for simulation with Abeta11-42 peptide (in the fibril-like form) binding to the fibrillar surface is shown. **6**

Figure S6. The free energy profile for an Abeta11-42 monomer (biased, fibril-like) binding to the fibril. **7**

Figure S7. Five key structural precursors of the fibril-like monomer on the fibrillar surface and their probability contact maps are shown. **8**

Figure S8. Probability contact maps for the five different structural precursors of the fibril-like monomer on the fibrillar surface are shown to a residue-to-residue resolution. **10**

Figure S9. The total twist angle of protofilaments of different sizes, averaged over three independent simulation trajectories, is shown with the error bar. **11**

Figure S10. The initial structure of the protofilament model of different sizes is analyzed and compared. **12**

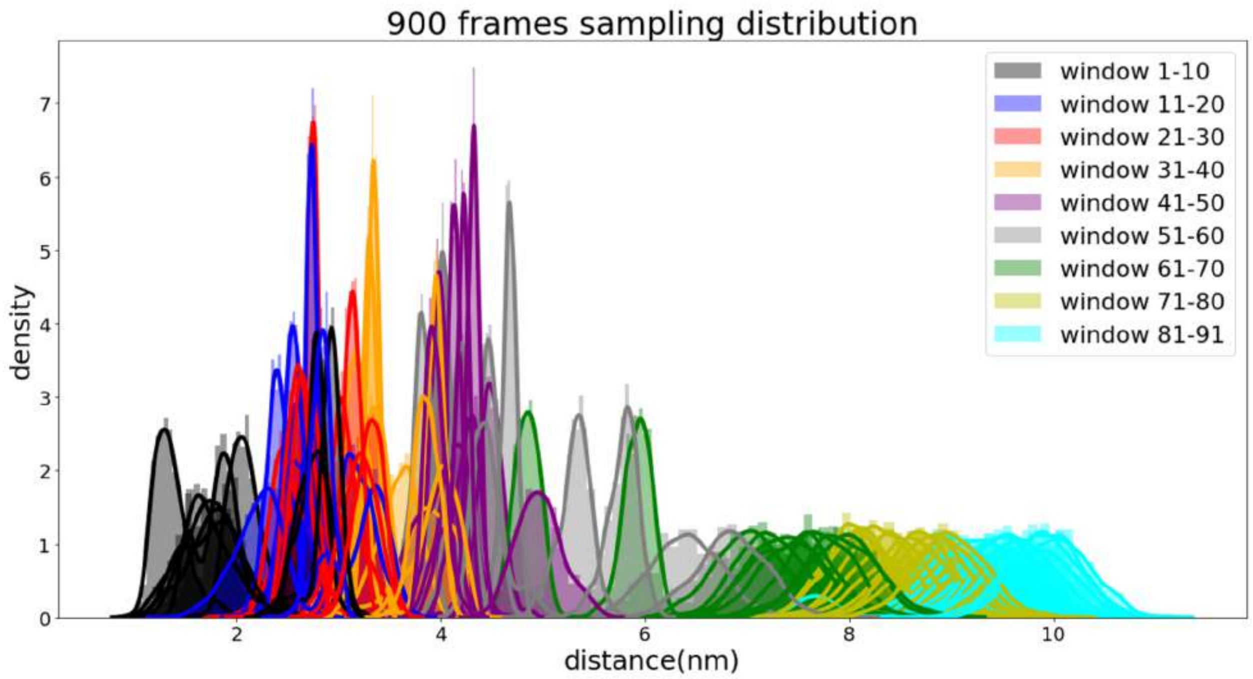

Figure S1. The distribution of sampling windows for simulation with Abeta11-42 peptide binding to the fibrillar surface is shown.

The Abeta monomer (unbiased), is put in the “front” orientation with respect to the central fibril. See the subplot in Fig.2(a) for all the six orientations. These sampling windows represent the last 900 frames of a total of 5000 frames. The data shown ensures that the free energy calculation is converged (See Fig.S2).

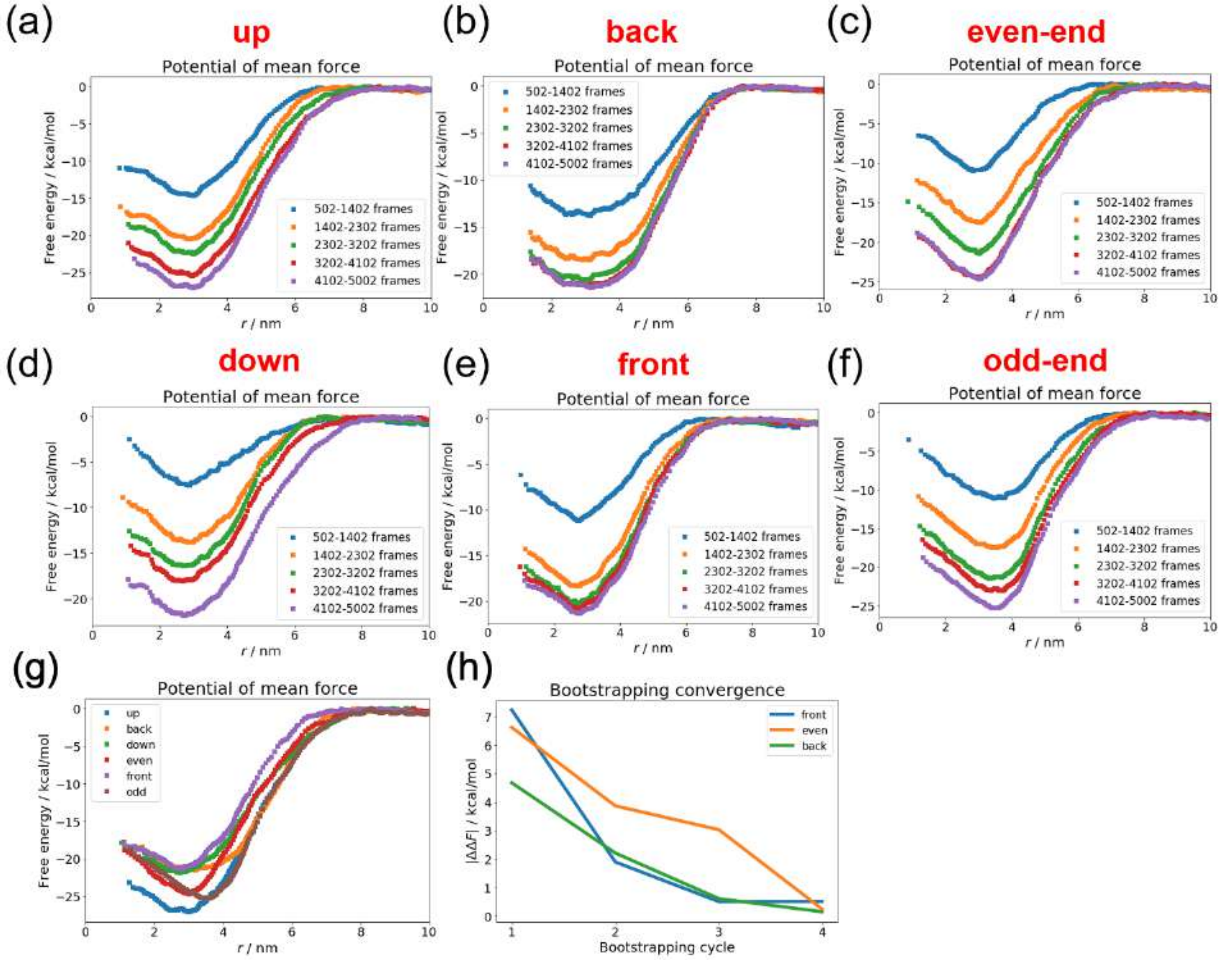

Figure S2. The free energy profile for an Abeta11-42 monomer (unbiased) binding to the fibril.

The simulation is carried out with the monomer being put in 6 different initial orientations, with respect to the central fibril (see subplot in Fig.2(a)). In each panel, a bootstrapping strategy is used to show the free energy profile every 900 frames. The first 500 frames are disregarded. (a) Up. (b) Back. (c) Even. (d) Down. (e) Front. (f) Odd. (g) Free energy profiles of all 6 orientations are shown together, only the last 900 frames are used for each. (h) Convergence test on the bootstrapping (|ΔΔF| < 0.6 kcal/mol).

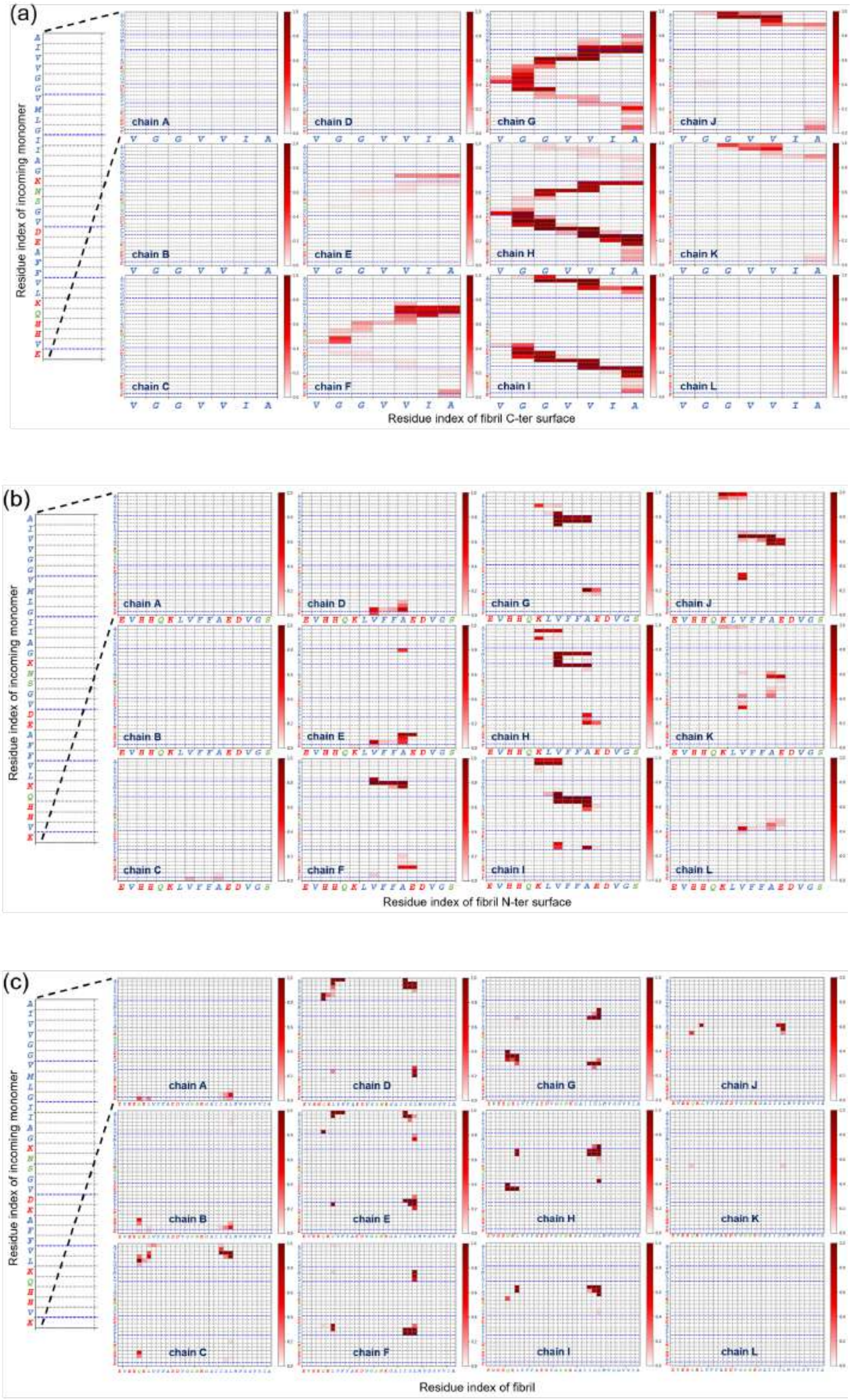
(a)
Residue index of incoming monomer
chain A
chain D
chain G
chain J
chain B
chain E
chain H
chain K
chain C
chain F
chain I
chain L
Residue index of fibril C-ter surface
(b)
Residue index of incoming monomer
chain A
chain D
chain G
chain J
chain B
chain E
chain H
chain K
chain C
chain F
chain I
chain L
Residue index of fibril N-ter surface
(c)
Residue index of incoming monomer
chain A
chain D
chain G
chain J
chain B
chain E
chain H
chain K
chain C
chain F
chain I
chain L
Residue index of fibril

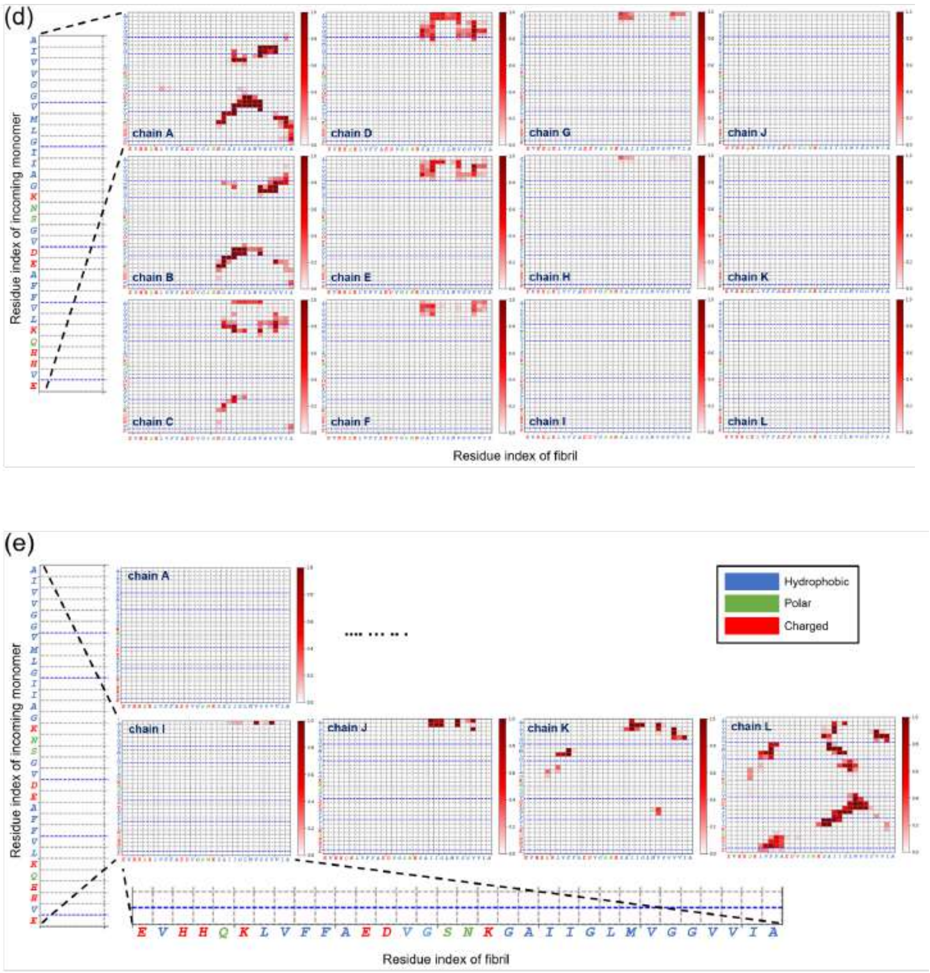

Figure S3. Probability contact maps for the five different structural precursors on the fibrillar surface are shown to a residue-to-residue resolution.

(a) C-ter surface precursor (b) N-ter surface precursor (c) Cleft-gate precursor (d) Even-end elongation precursor (e) Odd-end elongation precursor. The color bar scheme for the contact maps is the same as in Fig.3. Hydrophobic, polar, and charged residues are indicated in the sequence and are color coded. See the inset in (e).

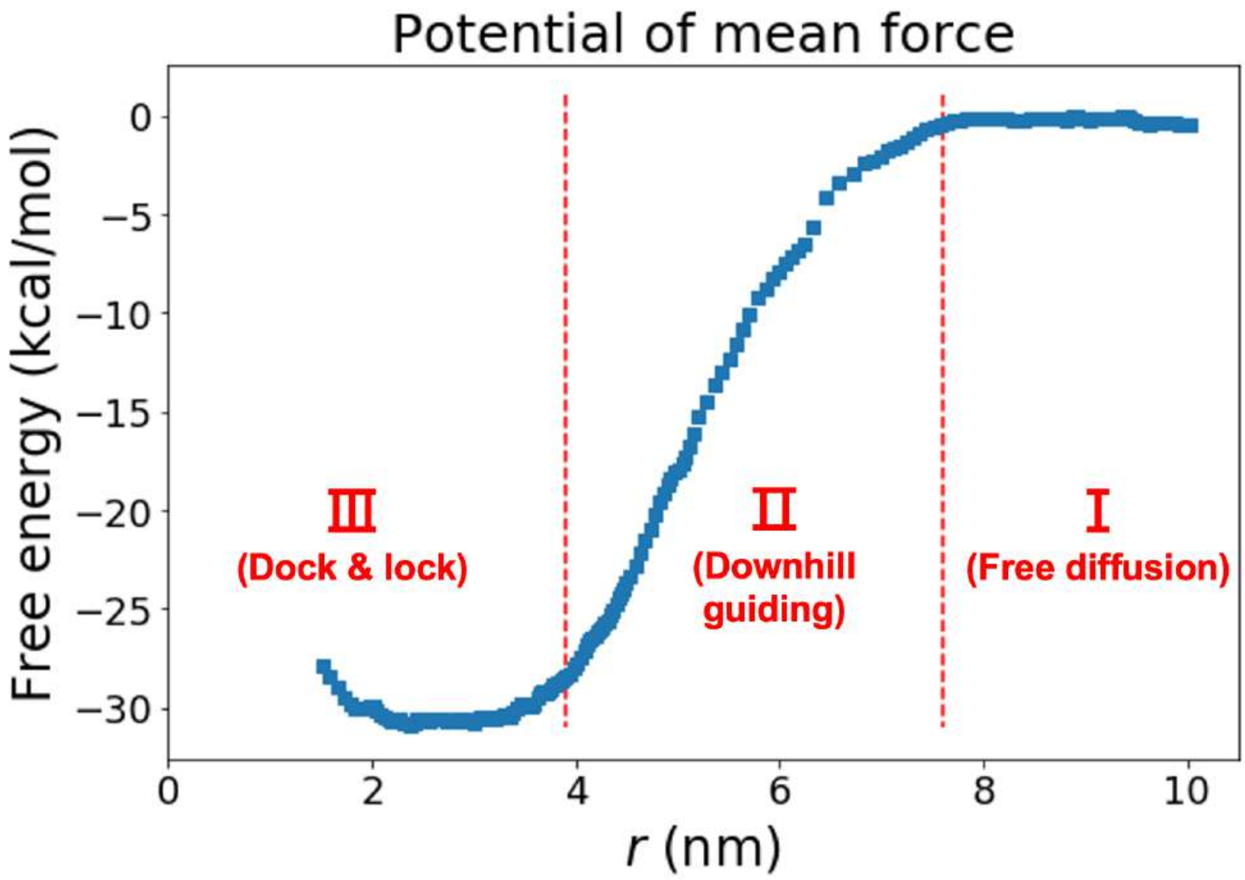

Figure S4. The free energy profile for a single fibril-like ensemble Abeta11-42 monomer binding to the Abeta42 fibrillar surface (12 chains) is shown (monomer in the ”back” orientation).

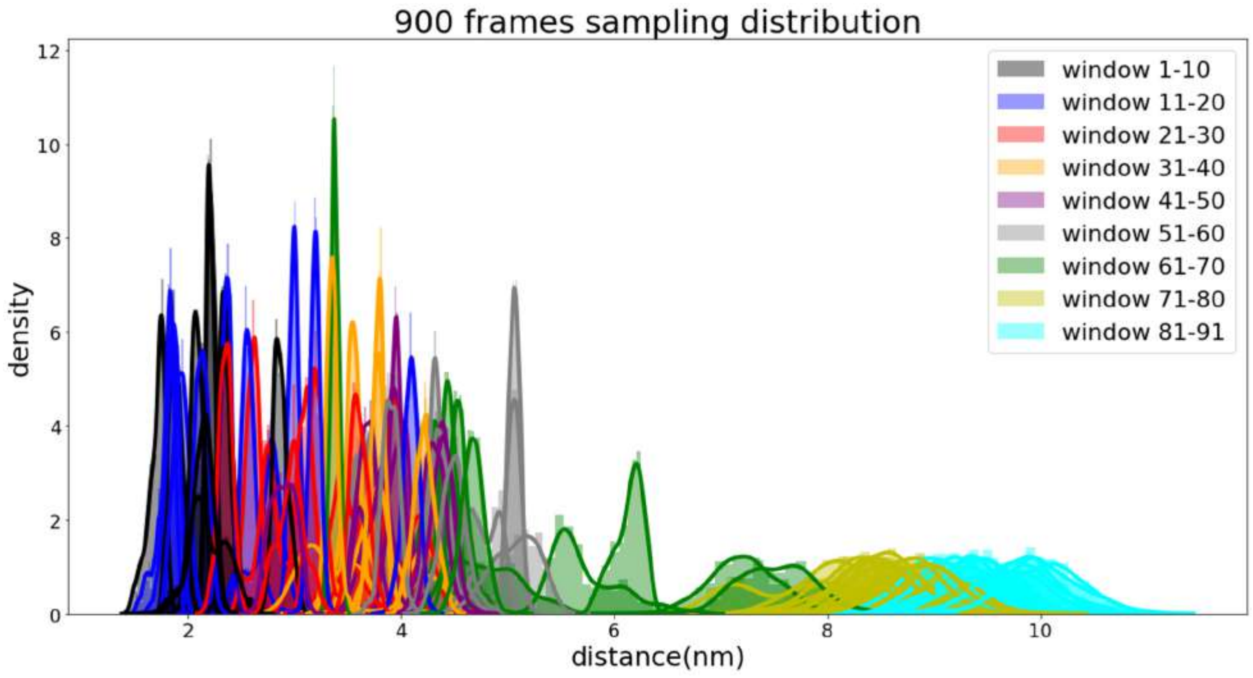

Figure S5. The distribution of sampling windows for simulation with Abeta11-42 peptide (in the fibril-like form) binding to the fibrillar surface is shown.

The Abeta monomer, biased in the fibril-like form, is put in the “back” orientation with respect to the central fibril. See the subplot in Fig.2(a) for all the six orientations. These sampling windows represent the last 900 frames of a total of 5000 frames. The data shown ensures that the free energy calculation is converged (See Fig.S6).

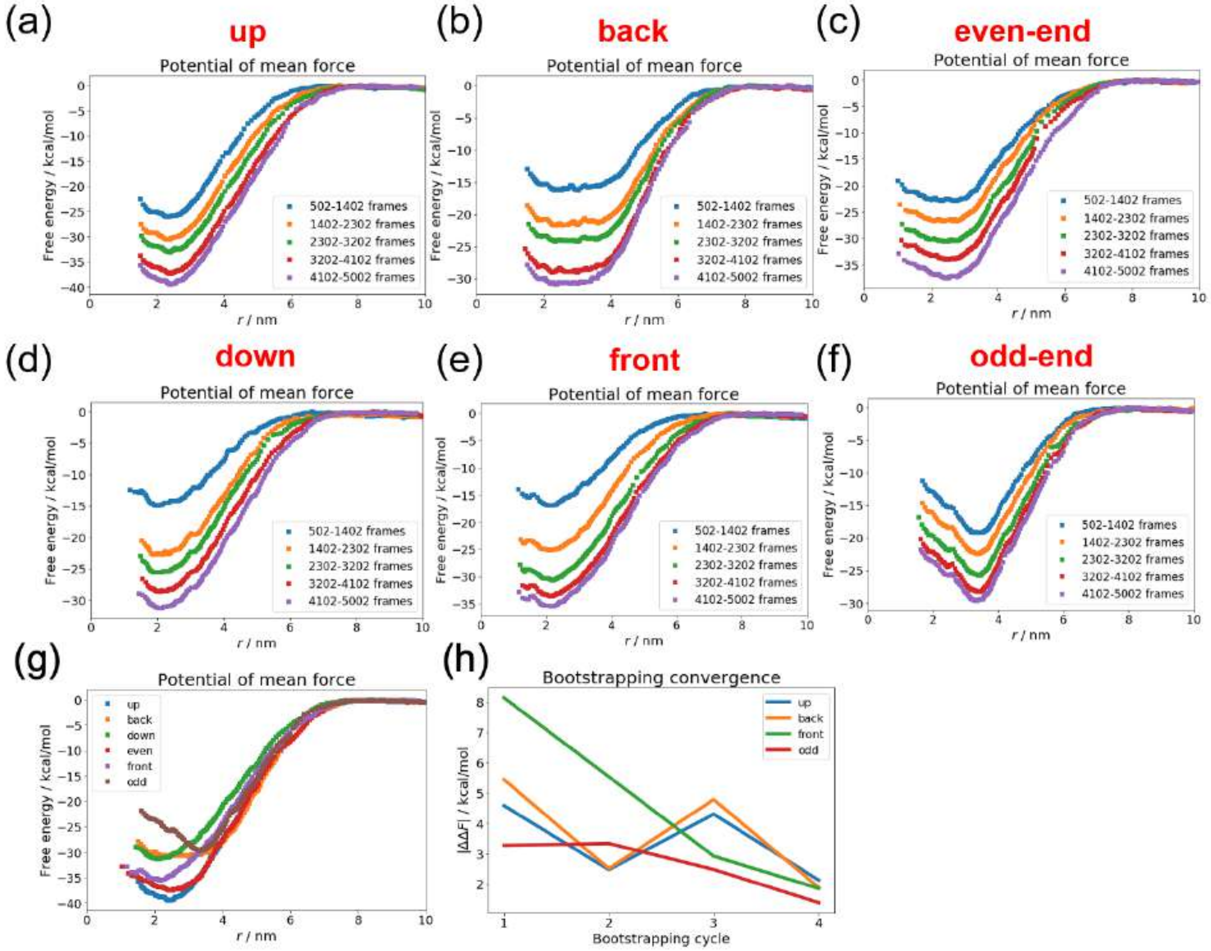

Figure S6. The free energy profile for an Abeta11-42 monomer (biased, fibril-like) binding to the fibril.

The simulation is carried out with the monomer being put in 6 different initial orientations, with respect to the central fibril (see subplot in Fig.2(a)). In each panel, a bootstrapping strategy is used to show the free energy profile every 900 frames. The first 500 frames are disregarded. (a) Up. (b) Back. (c) Even. (d) Down. (e) Front. (f) Odd. (g) Free energy profiles of all 6 orientations are shown together, only the last 900 frames are used for each. (h) Convergence test on the bootstrapping (|ΔΔF| < 0.6 kcal/mol).

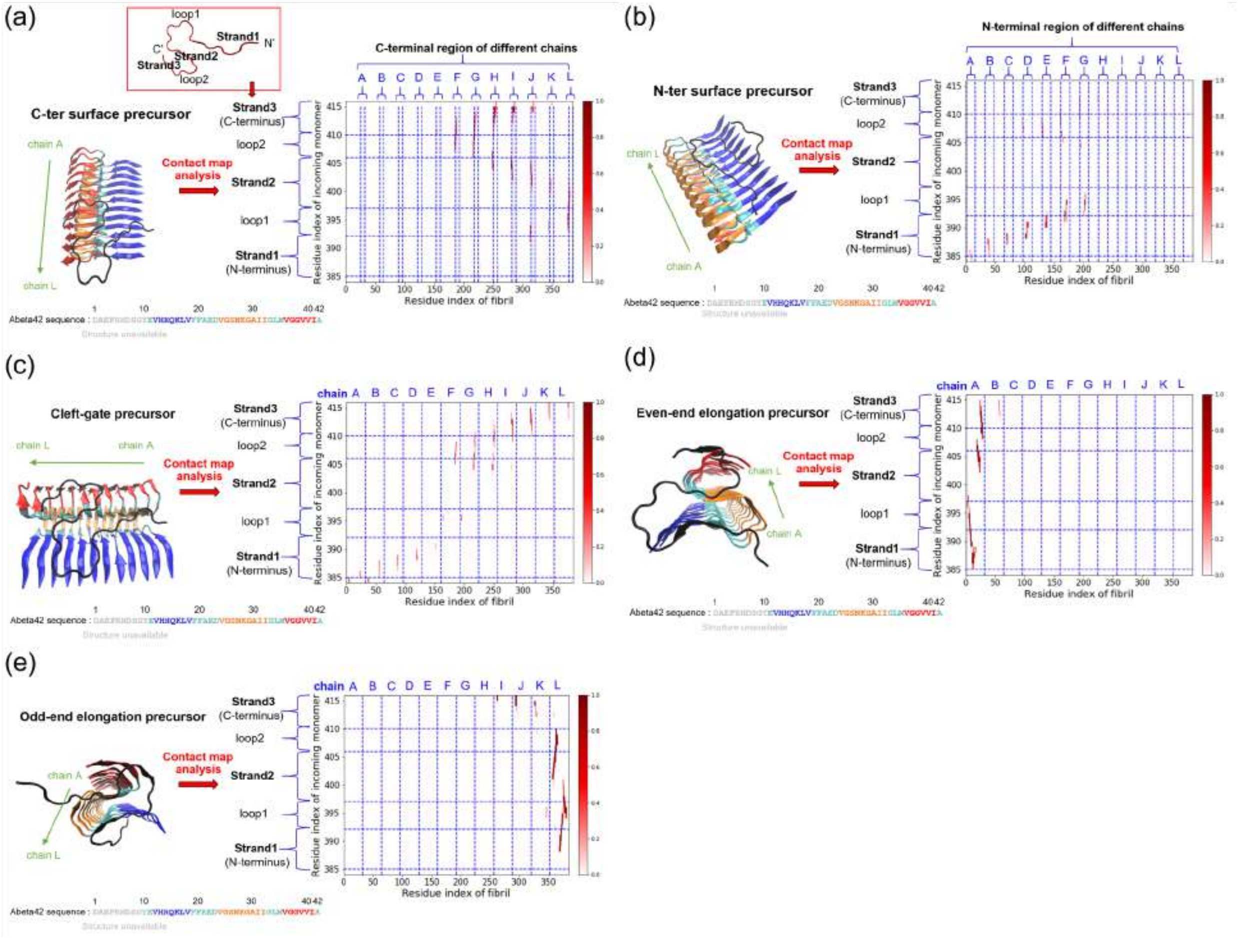

Figure S7. Five key structural precursors of the fibril-like monomer on the fibrillar surface and their probability contact maps are shown.

(a) C-ter surface precursor (b) N-ter surface precursor (c) Even-end elongation precursor (d) Odd-end elongation precursor. The probability contact map next to each structural precursor presents the contacts formed between the monomer and the fibril. The color bar, scaled by probability, is shown on the right. The horizontal axis uses the fibril index (1 to 384; 32x12=384), which sequentially renumbers the 12 monomers in the fibril. The vertical axis describes the residue index of the free monomer by adding up the existing fibril index, 385 to 416 (384+32=416). Different structural features are labeled as “strand” or “loop”. Note that in (a), a schematic monomer structure is shown in a red box, with its structure in the fibrillar form. Three strands (strands 1, 2, and 3) and two loops (loops 1 and 2) are indicated. The color scheme for the structure is the same as that in Figure 1.

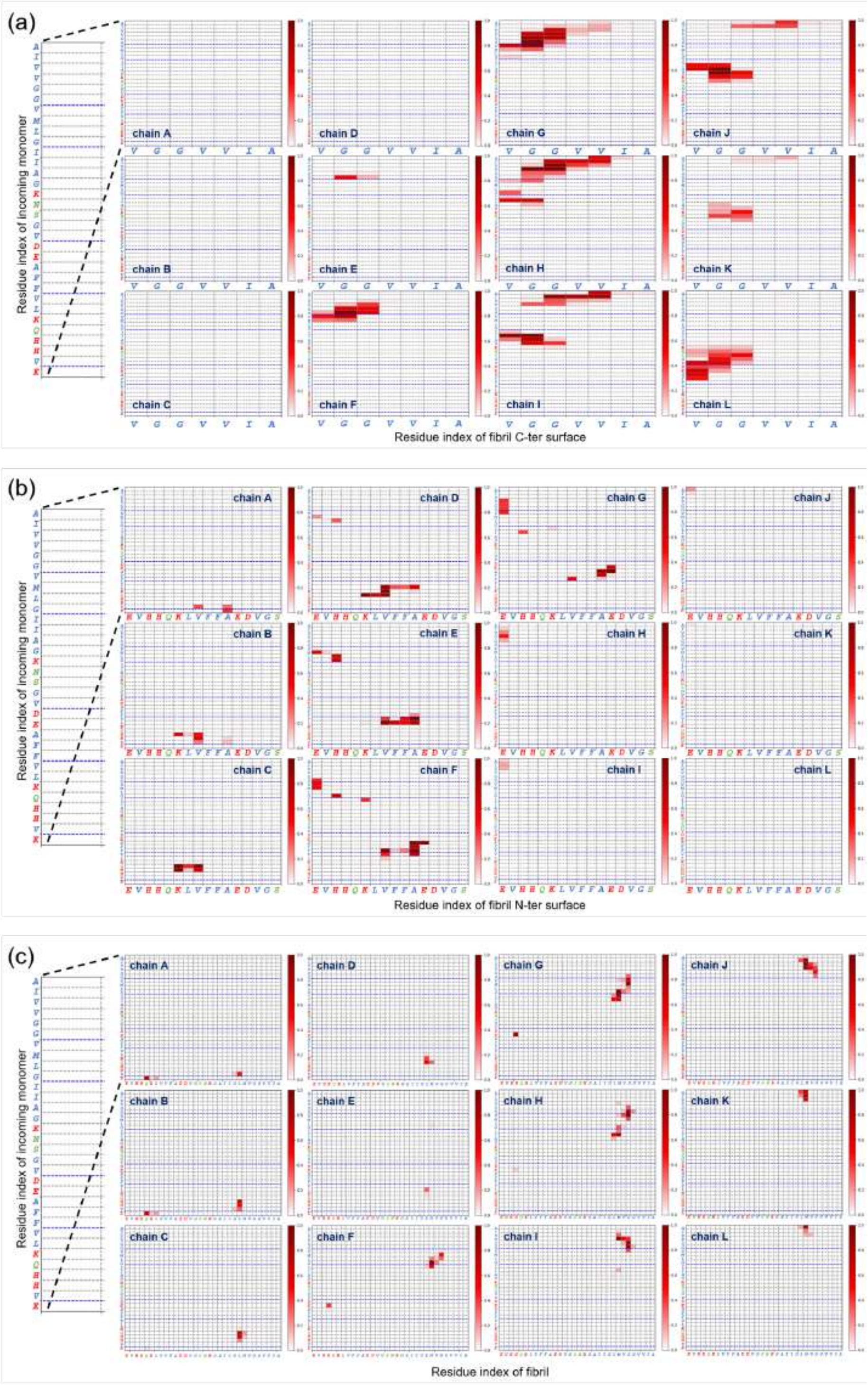
(a)
Residue index of incoming monomer
chain A
chain D
chain G
chain J
chain B
chain E
chain H
chain K
chain C
chain F
chain I
chain L
Residue index of fibril C-ter surface
(b)
Residue index of incoming monomer
chain A
chain D
chain G
chain J
chain B
chain E
chain H
chain K
chain C
chain F
chain I
chain L
Residue index of fibril N-ter surface
(c)
Residue index of incoming monomer
chain A
chain D
chain G
chain J
chain B
chain E
chain H
chain K
chain C
chain F
chain I
chain L
Residue index of fibril

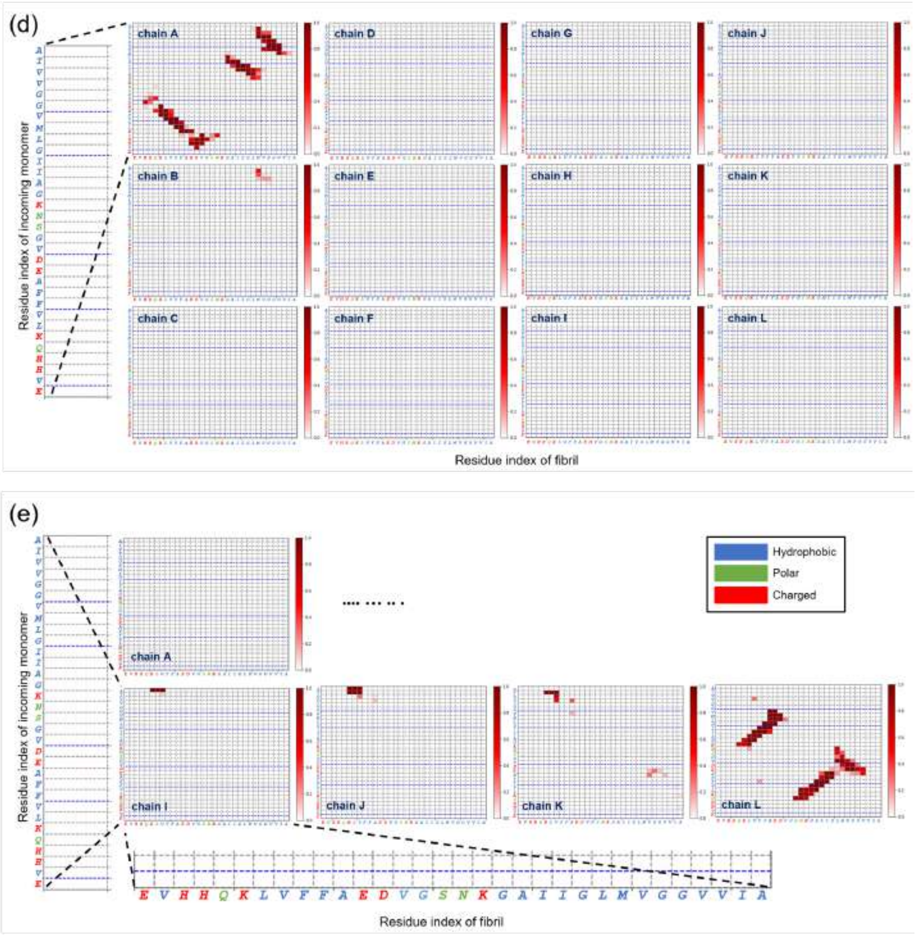

Figure S8. Probability contact maps for the five different structural precursors of the fibril-like monomer on the fibrillar surface are shown to a residue-to-residue resolution.

(a) C-ter surface precursor (b) N-ter surface precursor (c) Cleft-gate precursor (d) Even-end elongation precursor (e) Odd-end elongation precursor. The color scheme for the contact maps is the same as in Fig.3. Hydrophobic, polar, and charged residues are indicated in the sequence and are color-coded. See the inset in (e).

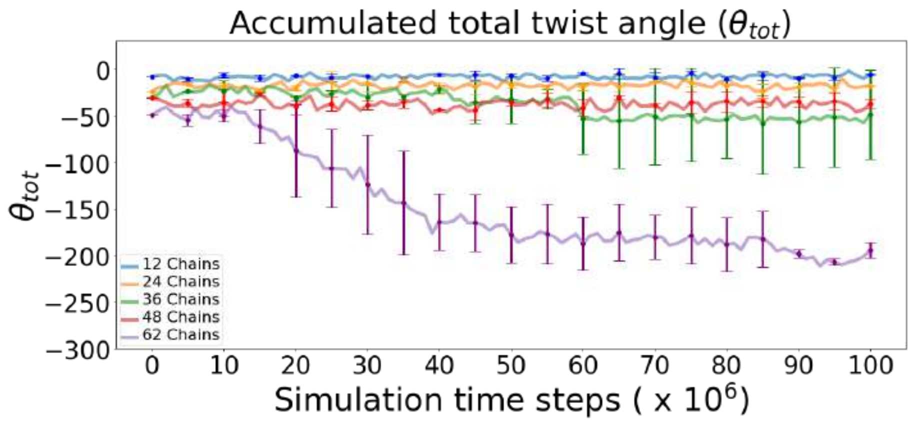

Figure S9. The total twist angle of protofilaments of different sizes, averaged over three independent simulation trajectories, is shown with the error bar.

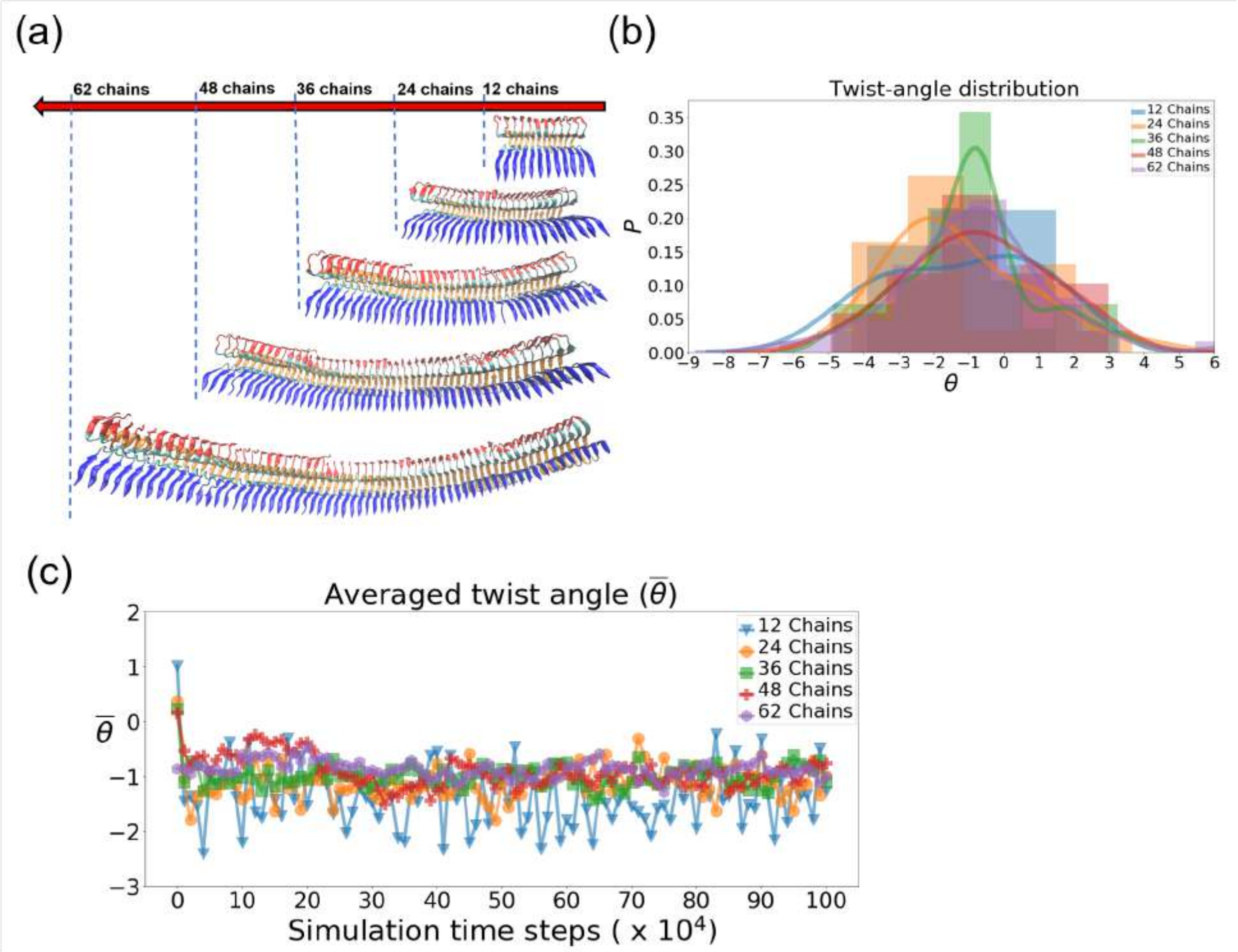

Figure S10. Initial structure of the protofilament model of different sizes is analyzed and compared.

(a) The initial fibrillar structures of different sizes are displayed (from top, 12, 24, 36, 48, and 62 chains). (b) Distribution of the twist angle (θ) for the initial structure. (c) Time evolution of the averaged twist angle (θ bar) in the pre-equilibration process, with the last frame representing the structure and angle distribution shown in (a) and (b), respectively.
